# Effectiveness of Yoga in Modulating Markers of Immunity and Inflammation: A Systematic Review and Meta-Analysis

**DOI:** 10.7759/cureus.57541

**Published:** 2024-04-03

**Authors:** Biswamohan Mishra, Ayush Agarwal, Jerry A George, Ashish D Upadhyay, Nilima Nilima, Rinkle Mishra, Neha Kuthiala, Aneesh Basheer, Venugopalan Y Vishnu, Vasantha Padma Srivastava

**Affiliations:** 1 Neurology, All India Institute of Medical Sciences, New Delhi, IND; 2 Biostatistics, All India Institute of Medical Sciences, New Delhi, IND; 3 General Medicine, Dr. Moopen's (DM) Wayanad Institute of Medical Sciences, Wayanad, IND

**Keywords:** il-6, tnf-alpha, inflammatory cytokines, systemic inflammatory markers, yogic breathing, yoga therapy

## Abstract

Chronic inflammation is central to the pathogenesis of many chronic inflammatory conditions. This review aims to analyze whether the practice of yoga, or yogic meditation and breathing, has any effect on the levels of inflammatory cytokines and other inflammatory markers in patients with various chronic inflammatory diseases such as rheumatoid arthritis, neoplastic disorders, and asthma, as well as in healthy subjects, compared to usual care or sham interventions. A comprehensive search of databases (PubMed, CENTRAL, Embase, and CINAHL) was performed. Randomized controlled trials (RCTs) that evaluated the effects of yoga as an intervention on inflammatory markers were analyzed. A total of 26 studies were included. Only two studies had a low risk of bias (RoB); 24 other studies had a high RoB. Most studies (n=24) reported a favorable outcome with yoga, irrespective of the type of yoga used, the condition studied, and the duration of the intervention. The commonly reported inflammatory markers included IL-6 (n=17), tumor necrosis factor-alpha* *(TNF-a) (n=13), and C-reactive protein (CRP) (n=10). Most studies showed a significant reduction in inflammatory markers in the yoga group (YG) compared to the control group (CG). Few studies also showed significant improvement in markers of cellular immunity (interferon gamma (IFN-g), IL-10, and transforming growth factor-beta (TGF-b); n=2 each) and improved mucosal defense (IgA, IL-6, and IL-2; n=2 each). A meta-analysis of IL-6, TNF-a, and CRP showed yoga had a favorable effect on the levels of these markers, but it was not statistically significant. Current evidence suggests that yoga can be a complementary intervention for various chronic inflammatory conditions. However, the quality of the evidence is poor, along with considerable heterogeneity. In the future, investigators should describe the intervention better, with a uniform assortment of outcome measures and treatment conditions, to generate high-quality evidence.

## Introduction and background

Yoga is a traditional Indian mind-body practice that is considered a way of life. The science and practice of yoga go beyond practicing *asanas*. Yoga is now being considered a healing design as well [1]. Regular and optimal practice of yoga is believed to be a natural immunity booster. Multiple chronic conditions, such as cardiac disease, carcinoma, rheumatoid arthritis, and allergic rhinitis, are characterized by indolent, persistent chronic inflammation [2-5]. Also, healthy individuals who are exposed to chronic stress have a higher risk of increased morbidity and mortality with age [6]. Chronic inflammation can be indirectly assessed by several biomarkers like IL-6, IL-10, IL-12, tumor necrosis factor-alpha (TNF-α), and by acute phase reactants like C-reactive protein (CRP), and total leukocyte counts [7]. Although results have been inconsistent, emerging evidence suggests that mind-body therapies may be able to lower levels of circulating pro-inflammatory cytokines [8,9].

The acceptance of yoga as a mind-body practice has grown in recent times. A recent survey reported that 34.4 million people in the US practice yoga, i.e., about 10% of the US population. The popularity of yoga grew by 68.3% from 2010 to 2021 [10]. Yoga originated in ancient India about 5000 years BC, and its major facets include postures (*asanas*), yogic breathing (*pranayama*), meditation (*dhyana*), concentration (*dharana*), and ecstatic union (*samadhi*), among others [11]. Existing research indicates a consistent downregulation of pro-inflammatory markers, notably IL-1 beta, with some evidence suggesting reductions in IL-6 and TNF-alpha, although results vary [12,13]. While inconsistencies exist, particularly regarding CRP, TNF-alpha, and IL-6, further investigation is warranted to confirm yoga's impact on these markers. Notably, yoga may exert anti-inflammatory effects on a transcriptional level, modulating nuclear factor kappa B (NF-kB) activity and gene transcription associated with anti-inflammatory responses [13,14]. Long-term practitioners exhibit lower pro-inflammatory marker levels, underscoring the importance of sustained practice. Moreover, yoga seems to enhance cell-mediated and mucosal immunity, potentially improving protection against pathogens [12]. However, research gaps persist, necessitating more studies to elucidate the effects of yoga across different populations and intervention durations.

Reviews till now have evaluated the potential effects of mind-body therapies on inflammatory markers. A review focusing on the potential effect of yoga only on chronic inflammation, apart from other mind-body therapies, is required to systematize the current evidence and determine the role of yoga in the clinical setting. Therefore, we systematically reviewed the available literature to analyze the effectiveness of yoga interventions on downregulating chronic inflammatory markers in individuals with various chronic inflammatory conditions as well as healthy individuals.

## Review

Methods

The research question in terms of population is: Does the practice of yoga or yogic meditation and breathing have any effect on the levels of inflammatory cytokines and other inflammatory markers in patients with various diseases and also healthy subjects compared to usual care or sham intervention? The participants included healthy individuals and those with any disease condition for which yoga was used as one of the interventions. This systematic review was performed per the Preferred Reporting Items for Systematic Reviews and Meta-Analyses (PRISMA) guidelines.

Interventions and Comparators

Studies with yoga interventions based on postures (asanas) of any overall duration, style, number of sessions, duration of sessions, and frequency of sessions were included. Yogic meditation and yogic breathing were also included. Studies comparing yoga to any other intervention or no intervention were also included. We also incorporated studies comparing yoga as an adjunct to other therapies, versus those other therapies, versus those other therapies alone. The comparisons of interest were: yoga vs. usual care (no intervention), yoga vs. sham intervention, and yoga plus any intervention vs. that intervention alone. Studies with cointerventions were allowed if the cointerventions were comparable between intervention groups.

Selection of Outcomes

All immune- and inflammation-related markers, such as immune-related cytokines and immune cells, were documented. We employed a two-step process for selecting outcomes. First, all available studies were reviewed to identify relevant outcomes within these categories. Subsequently, a consensus-based approach involving multiple reviewers was utilized to determine which of these outcomes would be included in the analysis. Those markers with no clearly established roles in immune and inflammation mediation were excluded.

The primary outcome parameters assessed were cytokines (IL-6, IL-1, TNF, and TNF-alpha). The secondary outcome parameters assessed were: other cytokines and circulating immune markers (IL-1beta, IL-8, IL-10, IL-12, interferon (IFN)-gamma, IL-12, soluble IL-2 receptor (sIL-2r), soluble TNF-receptor II (sTNFrII), and circulating endothelial microparticles (EMPs); regular inflammatory markers (CRP, total leucocyte count, eosinophils, lymphocytes); antiviral-related immune outcomes (IFN-gamma, natural killer (NK)-cytotoxicity, CD4, CD8, NK cells); antibody responses (IgA); and markers of gene expression (pro-inflammatory transcription factors NF-kB and CREB family transcription factors).

Study Design

The present study is a systematic review and meta-analysis involving a comprehensive analysis of existing literature. Randomized controlled trials (RCTs) that evaluated the effects of yoga as an intervention on the immune system and/or its functioning were included in this review. The RCTs with human participants, irrespective of age, gender, and underlying clinical condition, were included. Those RCTs comparing the effects of yoga as an intervention for at least one parameter of the immunological system versus no yoga or usual standard of care were also included. The outcomes assessed were various immunological markers such as immune cell counts, cytokines, antibodies, and markers of genetic expression. Systematic reviews, cohort studies, case reports, case-control studies, policy papers, letters to the editor, correspondence, and non-human studies were excluded. Trials in which the results were confounded by a treatment or a control group receiving another active treatment that had not been considered in the randomization were also excluded.

Quality of Evidence

The risk of bias (RoB) was assessed independently by two assessors, BM and AA, using the revised Cochrane risk-of-bias tool for randomized trials (RoB2) [15]. Using this tool, the RoB can be evaluated using five different domains. Each domain begins with one or more signaling questions, which the assessors need to mark as yes (Y), no (N), probably yes (PY), probably no (PN), and no intervention (NI). The assessor's judgment in each domain finally leads to an overall judgment of “low risk of bias,” “high risk of bias,” or “some concerns.”.

Search Strategy 

A comprehensive search (see Appendix A for the search terms) was conducted on the Cochrane Central Register of Controlled Trials (CENTRAL), MEDLINE via PubMed (1990 to October 2022), Embase (1980 to October 2022), and the Cumulative Index to Nursing and Allied Health Literature (CINAHL; 1982 to October 2022). The reference lists of all the studies retrieved were searched for any missing papers. No restrictions were placed concerning language or publication status. The primary search terms used were 'yoga', 'yogic meditation', 'yogic breathing', 'immune', and 'immune markers. The studies that appeared in the search and met the above-mentioned selection criteria were included for further review. In cases of the non-availability of full texts, the corresponding authors were contacted for full texts.

Two independent reviewers (BM and AB) screened all citations based on the title or abstract. All full-text articles were read by BM, and 10% were read by reviewers AA and JAG. Any disagreements were discussed with a third reviewer (VYV or RM) to reach a consensus. Articles were categorized as part of the same cohort when there was evidence of shared recruitment sites, study dates, and grant funding numbers, or when they reported similar or identical patient characteristics unless explicitly stated otherwise in the publications.

Data Extraction

Data extraction was done using Covidence Extraction 2.0 (Veritas Health Innovation, Melbourne, VI, AUS) [16]. For each outcome, data were collected at baseline (before intervention) and post-intervention in terms of mean and standard deviation or standard error of the mean, median, inter-quartile range (IQR), mean differences, and confidence interval (CI) with effect size from the information available in the studies. Studies reporting multiple time points were also collated. To address the issue of multiple time points in the studies, we opted to extract data from the last available time point or, when available, the maximum duration of pre- and post-intervention measurements. Data were categorized based on sample size, baseline characteristics, type of yoga, duration and supervision, dropouts, adverse events, a summary of major results, and major limitations. The final accumulation and interpretation of the results were made by authors BM and AA.

Data Analysis

Although yoga or its modified version was a common modality of intervention in the active arm in all the studies, there was considerable heterogeneity in the duration of yoga, its components, the duration and timing of outcome assessment, and the outcomes assessed. The available data is mostly presented in a descriptive, systematic manner in this review. Only those outcome measures for which data was available in four or more studies were included in the meta-analysis.

Estimating the Total Duration of Exposure to Yoga

The studies reported the duration of yoga in terms of minutes, days, weeks, and months. For uniformity, the total dose of yoga for each study was expressed in terms of minutes. The session length was multiplied by the number of times it was administered per week by the total number of weeks to obtain the total duration of exposure of the participants to yoga in each study. In the majority of the studies (n=17), the yoga was supervised by a certified yoga instructor, while in others, instructions were provided for home-based yoga either in a brochure, online platform, or audio video compact disk (CD).

Medical and Surgical Conditions Included in the Studies

Five studies evaluated participants with cancer, three evaluated breast cancer survivors [17-19], one evaluated colorectal cancer [20], and another evaluated myeloproliferative neoplasms (MPN) [21]. Four studies evaluated participants with rheumatoid arthritis [22-25]. Healthy volunteers recruited by the various studies consisted mainly of student volunteers in four studies [26-29] and female Chinese subjects in one study [30]. Two studies each evaluated the effect of yoga on patients with heart failure [31,32] and healthy adults exposed to high-risk occupational hazards [33,34]. One study each enrolled healthy pregnant women [35], family dementia caregivers [36], patients with asthma [37], healthy subjects with chronic periodontitis and Indian adults with metabolic syndrome [38], patients with allergic rhinitis [39], type 2 diabetes mellitus [40], and major depressive disorder [41]. The majority of the studies received funding for their research, except for three studies that received no funding and four that provided no information on funding.

Possible Conflicts of Interest in the Included Studies

In the study by Black et al., there was a possible conflict of interest, wherein the corresponding author received grant funding from the Forest Research Institute and was a consultant to the Lilly and Dey Pharmaceutical Companies at the time of manuscript publication [36]. Two others had not mentioned any information regarding any conflict of interest [32,25]. The rest of the studies had no conflict of interest among the authors or funding agencies.

Statistical Analysis

The data synthesis was done in a systematic manner per the planned parameters. The meta-analysis was conducted using R version 3.6.2 (R Project for Statistical Computing, R Core Team, Vienna, Austria). The pooled prevalence and 95% CI were calculated. The prevalence estimates were transformed using the Freeman-Tukey double arcsine transformation. The reporting and publication biases were assessed using Begg’s funnel plot and Egger’s regression test. The heterogeneity of the intervention effect in various included studies was assessed using the standard chi2 statistic (p-value) or the I² statistic. A p-value >0.10 was considered statistically significant heterogeneity. A fixed-effect model was used for I² <40%; otherwise, the results were combined using a random-effect model. The p-value <0.05 was considered statistically significant.

Results

Study Selection

**Figure 1:**
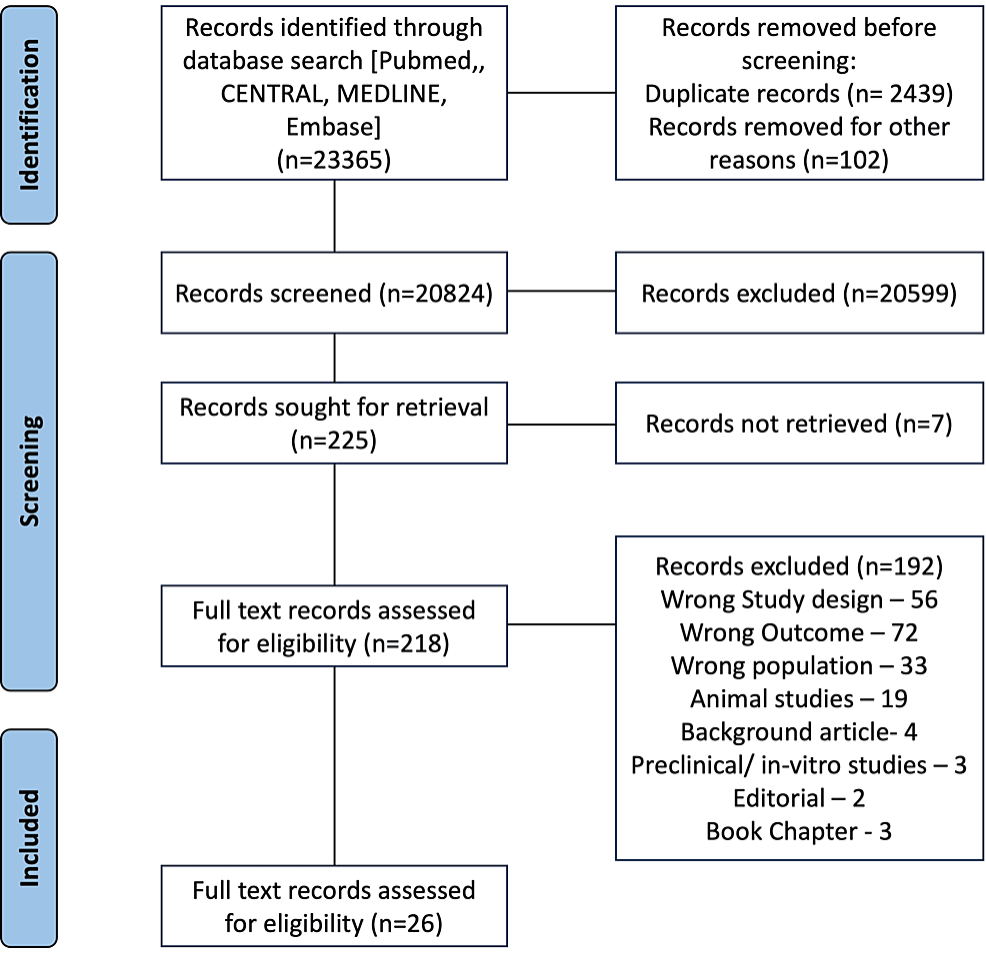
PRISMA flowchart depicting the identification, screening, and inclusion of studies for this systematic review PRISMA: Preferred Reporting for Systematic Reviews and Meta-analyses

A total of 23,365 references were imported into Covidence for screening as 23,365 studies, from which 2439 duplicates were removed. Then, 20,824 studies were screened against title and abstract, and 20,599 studies were excluded. A total of 225 studies were sought for retrieval; full test records for seven studies could not be retrieved; 218 studies were assessed for full-text eligibility; and 192 studies were excluded. Finally, 26 studies were included in this systematic review. Figure 1 shows a PRISMA flow diagram depicting the above-mentioned screening and selection process. For this review, the yoga group is termed YG, and the control group is termed CG. 

Quality of Evidence and Risk of Bias Assessment

The risk of bias was assessed independently by two assessors, BM and AA, using the RoB2 tool for randomized trials (see Appendix B) [15].

Characteristics of the Included Studies

Participants and setting: A total of 26 studies were included in this review. A total of 2449 subjects were enrolled, out of whom 2091 had data available for analysis. The overall drop-out rate was 14.6%. The sample size varied from 15 [20] to 276 subjects [37]. In the YG, 418 of 645 participants were females (64.8%), and in the CG, out of 672, there were 408 female participants (60.7%). Details of other baseline characteristics of the studies included are provided in Table 1. There was considerable heterogeneity in the reporting of the baseline characteristics of the participants. One study did not report any baseline characteristics of the enrolled subjects [42].

**Table 1:** Demographics, setting of the study, and baseline characteristics of the trials included in this systematic review BDI: Beck depression inventory score, DAS 28 ESR: Disease activity score in 28 joints, DBP: Diastolic blood pressure, DM: Diabetes mellitus, HAM D: Hamilton rating scale for depression, HDL: High-density lipoprotein, HR: Heart rate, HTN: Hypertension, LDL: Low-density lipoprotein, MMSE: Mini-mental state examination, NI: No information, NA: Not applicable, OHA: Oral hypoglycaemic agent, RA: Rheumatoid arthritis, RR: Respiratory rate, SBP: Systolic blood pressure, SF-36 MCS: Short form 36 mental health composite summary score, SF-36 PCS: Short form 36  physical health composite summary score, SSRI: Selective serotonin reuptake inhibitor, TG: Triglyceride

No.	Author and year of publication	Study population	Total participants enrolled, and total that completed the study	Type of intervention in the cases arm	No. of subcomponents	Name of subcomponents	Duration x frequency per week x no. of weeks = total duration in minutes	Presence of interventions in the control group	Age for YG, and CG	Female gender depicted as n (%) for YG, and CG	Severity of the disease and characteristic of the condition under study for YG, and CG
1	Kiecolt-Glaser et al., 2014 [17]	Breast cancer survivors	200, 186	Hatha yoga	3	Asanas, pranayama and meditation	90 x 2 x 12 = 2160	No. Usual daily routine with waitlisted controls	51.8 ± 9.8, 51.3 ± 8.7	NI	YG: Cancer stages 0 = 9 (9%); I = 46 (46%); IIA = 27 (27%); IIB = 10 (10%); IIIA = 8 (8%), CG: Cancer stages 0 =9 (9%); I = 43 (43%); IIA = 25 (25%); IIB = 13 (13%); IIIA = 10 (10%)
2	Black et al., 2013 [36]	Family dementia caregivers	45, 39	Kirtan kriya meditation (KKM) – incorporated from kundalini yoga practice	2	Yogic meditation and breathing exercises	12 x 7 x 8 = 672	Yes, relaxing music (RM)	60.5 ± 28.2, 60.6 ±12.5	23 (100), 14 (88)	YG: Distress, HAM-D = 11.9 ± 4.1; cognition, MMSE = 29.5 ± 1.0; SF-36, CG: Distress, HAM-D = 11.4 ± 4.0 cognition, MMSE = 29.6 ± 0.6
3	Mahendra et al., 2017 [42]	Healthy subjects with chronic periodontitis	60, 60	Pranayama	1	Breathing oxidative	20 x 7 x 12 = 1680	No. Standard therapy (SRP)	NI	NI	NI
4	Chanta et al., 2021 [39]	Allergic rhinitis	30, 27	Hatha yoga	3	Relaxation, asanas, and mediation	60 x 3 x 8 = 1440	No. Usual daily routine	NI	10 (77), 11 (78.6)	NI
5	Yadav et al., 2019 [38]	Indian adults with metabolic syndrome	260, 168	Yoga-based lifestyle interventions (YBLI)	5	Asanas, pranayama, cleansing exercises, and meditation.	120 x 7 x 12 = 10,080	Yes, dietary interventions	NI	NI	YG: Waist circumference (cm) = 97.8 ± 11; HDL-C (mg/dl) = 44.7 ± 4.9; triglyceride (mg/dL) = 128.5 (98.0, 174.5), CG: Waist Circumference (cm) = 96.7 ± 11; HDL-C (mg/dl) = 45.6 ± 4.6; triglyceride (mg/dL) = 133.5 (94.8, 170.3)
6	Singh et al., 2011 [22]	Rheumatoid arthritis (RA)	80, 80	Yoga	4	Asanas, cleansing practice pranayama, meditation, and a healthy diet	90 x 6 x 7 = 3780	No. Standard medical therapy, and waitlisted controls	35.07±7.3, 34.65±7.3	NI	YG: Pain intensity = 1.90± 0.84; inflamed joints = 3.38± 1.31; morning stiffness = 81.68± 21.65, CG: Pain intensity = 2.03±0.7; inflamed joints = 3.25± 1.24; morning stiffness = 76.25± 20.56
7	Gopal et al., 2011 [26]	Student volunteers	60, 60	Integrated yoga practices	3	Asanas, pranayama, and meditation	35 x 7 x 12/ 2940	No. Usual daily routine	NI	NI	NA
8	Lim et al., 2015 [27]	Student volunteers	25, 25	Yoga practice	4	Pranayama, awareness exercises, asanas, and meditation	90 x 1 x 12 = 1080	No. Normal daily routine	21.0 (19.0–23.0), 22.0 (19.0–25.0)	6(50), 8 (61.5)	NA
9	Fujisawa et al., 2018 [28]	Student volunteers	120, 120	Laughter yoga	3	Laughter and yogic breathing, meditation	30 (one session) = 30	Yes, comedy movie group and reading book group	24.4± 3.8, 24.0 ± 3.6	14 (35), 16 (40)	NA
10	Chen et al. 2017 [35]	Healthy pregnant women	101, 94	Yoga	4	Physical postures/stretching, deep breathing, guided imagery, and deep relaxation	70 x 2 x 20 = 2800	No. Routine prenatal care	33.1 ± 4.02, 33.1 ± 4.02	48 (100), 46 (100)	YG: Gestational age (weeks) =39.3 ± 0.71; birth weight = 3170.5 ± 240.54 in grams, CG: Gestational age (weeks) = 38.6 ± 1.32; birth weight = 3048.2 ± 269.67 grams
11	Agnihotri et al., 2014 [37]	Asthma	276, 240	Yoga intervention	3	Asanas, pranayama, and meditation	13 x 5 x 24 = 1560	No. Standard care	37.03±11.46, 38.69±10.54	54 (45), 49 (40.83)	YG: Mild = 100 (82.64%); moderate = 21 (17.36%), CG: Mild =96 (80%); moderate = 24 (20%)
12	Twal et al., 2016 [29]	Student volunteers	20, 20	Yogic breathing	1	Yogic breathing (pranayama)	20 (one session) = 20	Yes, attention control (AC)	33, 29	5 (50), 5 (50)	NA
13	Sohl et al., 2016 [20]	Colorectal cancer	15, 11	Yoga skills training	4	Awareness meditation, movement, breathing, and relaxation	15 x 4 x 8 = 480	Yes, attention Control	61.0 (44.0 – 67.0)(combined YG+CG)	6 (40) (combined YG+CG)	YG + CG: Colon cancer n (%) = 10 (67%); rectal cancer n (%) = 5 (33%); stage at diagnosis 0–II = 4 (27%), III = 6 (40%), IV = 5 (33%) (overall)
14	Pullen et al., 2008 [32]	NYHA class I-III heart failure patients	19, 19	Yoga therapy	3	Asanas, pranayama, and meditation	70 x 2 x 8 = 1120	No. Standard medical therapy	52.1 ± 3.3/50.5 ± 12.8	7 (77.8), 3 (30.0)	YG: New York Heart Association Class (NYHA) n (%) = I – 3 (33.3); II – 3 (33.3); III – 3 (33.3), CG: NYHA n (%) = I – 4 (40.0); II – 2 (20.0); III – 4 (40.0)
15	Huberty et al., 2019 [21]	Myeloproliferative neoplasms (MPN)	56, 48	Online streamed yoga	4	Breathing, movement, poses, and meditation	60 x 1 x 12 = 720	No. Usual routine care and waitlisted control	58.3 ± 9.3, 55.0 ± 11.4	25 (92.6), 20 (95.2)	NI
16	Pullen et al. 2010 [31]	Systolic or diastolic HF of ischemic or nonischemic etiology.	40, 34	Yoga	4	Asanas, pranayama, breathing exercises, relaxation	60 x 2 x 8 = 960	No. Standard medical care	55.8± 7.6, 52.5 ± 12.7	NI	YG: NYHA class, n (%) - I = 6 (29); II = 8 (38); III = 7 (33), CG: NYHA class, n (%) - I = 5 (26) ; II = 8 (42) ; III = 6 (32)
17	Gautam et al., 2020 [24]	RA	66, 62	YBLI	4	Yogasana, relaxation, pranayama, and meditation	120 x 5 x 8 = 4800	No. Standard medical therapy	45.1± 8.7, 43.4 ± 9.3	28 (84.8), 25 (75.8)	YG: DAS28-ESR = 5.2 ±1.1, CG: 5.0 ± 0.8
18	Nugent et al., 2021 [41]	Major depressive disorder	87, 87	Hatha yoga	4	Pranayama, asanas, relaxation, and meditation	80 x 2 x 10 = 1600	Yes, health education	45.52 ± 12.72, 44.79 ± 13.79	44 (91.7), 29 (74.36)	YG: Baseline depression score = 12.67± 2.83, CG: Baseline depression score =1 2.87 ± 2.70
19	Shete et al., 2017 [33]	Healthy male adults with high-risk occupational hazards	48, 37	Yoga	2	Asanas and pranayama	60 x 6 x 12 = 4320	No. Waitlist controls	30 to 58 years (41.5 ± 5.2) combined YG+CG	NI	YG: Cholesterol (mg/dl) = 170.0 ±23.6); triglyceride (mg/dl) =123.6 (69.7); HDL (mg/dl) =39.1 ± 3.8, CG: Cholesterol (mg/dl) =180.4± 26.0; triglyceride (mg/dl) =134.8 ± 58.2; HDL (mg/dl) =41.0 ± 2.9
20	Chen et al., 2016 [30]	Healthy, lean, and female Chinese subjects	30/30	Hatha yoga	4	Asanas, relaxation, breathing, and meditation	60 x 2 x 8 = 960	No. Usual daily routine	NI	15 (100), 15 (100)	YG: HDL-C (mM) = 1.67 ± 0.05; LDL-C (mM) = 2.14 ± 0.11; cholesterol (mM) = 4.13 ± 0.12, CG: HDL-C (mM) = 1.93 ± 0.15 LDL-C (mM) = 3.90 ± 0.18; cholesterol (mM) = 0.60 ± 0.06
21	Rajbhoj et al., 2015 [34]	Healthy industrial workers with high risk of occupational hazards	48, 37	Yoga practice	3	Asanas, relaxation, and meditation	45 x 6 x 12 = 3240	No. Usual daily routine and waitlisted control	40.72 ± 6.79, 40.18 ± 6.31	NI	NA
22	Bower et al., 2014 [18]	Stage 0-II breast cancer survivors having cancer-related fatigue	31, 28	Iyengar yoga (a type of hatha yoga)	2	Postures and breathing techniques	90 x 2 x 12 = 2160	Yes, health education classes	54.4 ± 5.7, 53.3 ± 4.9	NI	YG: SF-36 vitality score = 37.8 (16), CG: SF-36 vitality score = 34.0 (16.3)
23	Parma et al., 2015 [19]	Previous invasive breast cancer or ductal carcinoma in-situ	94, 72	Yoga	NI	NI	60 x 3 x 24 = 7560	Yes, comprehensive exercise (CE) and comparative exercise (C)	56.7 ± 9.6/57.6 ± 6.6	NI	NA
24	Ganesan et al., 2020 [23]	RA	166, 143	Yoga	4	Asanas, pranayama, relaxation, and meditation	30 x 3 x 12 = 1080	No. Standard medical therapy	41.33 ± 9.51, 42.59 ± 7.1	63 (92.6), 68 (90.67)	YG: DAS28 = 4.95 ± 0.74, CG: DAS28 = 4.77 ± 0.62
25	Viswanathan et al., 2021 [40]	Type 2 diabetes	400, 300	Yogasanas using a yoga module	3	Asanas, relaxation, and pranayama	30 x 5 x 12 = 1800	Yes, simple physical exercises	50.8 ± 8.3, 52.8 ± 7.0	47 (31), 57 (38)	YG: Duration of DM (years) = 7.2 ± 5; TG (mg/dl) = 123 ± 6; HDL-C (mg/dl) = 40 ± 13, CG: Duration of DM (years) = 8.3 ± 5.8; TG (mg/dl) = 119 ± 74.5; HDL-C (mg/dl) = 40 ± 12
26	Gautam et al., 2019 [25]	RA	72, 64	Yoga-based mind-body interventions	4	Asanas, relaxation, meditation, and pranayama	120 x 5 x 8 = 4800	No. Usual standard care	45.7 ± 1.6, 42.1 (1.7)	29 (80.6), 28(84.8)	YG: DAS28-ESR = 5.13±0.2; HAQ-DI = 0.59 ± 0.06; BDI-II score = 19.14 ± 1.0, CG: DAS28-ESR = 4.95 ± 0.1; HAQ-DI = 0.53 ± 0.06; BDI-II score = 18.33 ± 0.9

Yoga design and interventions: In the studies included, some had named the type of yoga, some had mentioned yoga as part of a module for mind-body and lifestyle interventions, and some used the term 'yoga' only to describe the intervention. Among the named yoga practices, Hatha yoga was the most common type of yoga used. Five studies [17,39,41,30] used Hatha yoga, including one with Iyengar yoga [18], which the authors considered a type of Hatha yoga. Two studies each used yoga-based lifestyle and mind-body interventions [24,25,38] and pranayama only, i.e., yogic breathing [42,29] as interventions in the YG. One study used kirtan kriya meditation (KKM) derived from kundalini yoga practice [36], and another used laughter yoga (LY) [28]. Fifteen other studies had mentioned the style of yoga as 'yoga' only, 'yoga practice', 'yoga therapy’, or ‘integrated yoga’.

Constituents of yoga intervention: Yogic postures, or asanas, were considered distinct from physical exercises and limb movements. Similarly, yogic breathing was used synonymously with pranayama but was distinguished from simple breathing exercises for reporting results in this review. The number of sub-components of the yoga intervention employed ranged from one to five, with a median IQR of 3 (3,4) subcomponents. Asanas were an integral part of the yoga intervention in most of the studies (n=20), followed closely by yogic breathing, or pranayama, and meditation in 17 studies each. Eleven studies used relaxation techniques, and nine studies incorporated other breathing techniques or exercises (not labeled as pranayama or yogic breathing in these studies) as part of the yoga module. Laughter yoga was used in one study [28]. Some studies used additional subcomponents like cleansing exercises [38,22] and dietary counseling (Table 1) [22].

Type of intervention in controls: The control conditions used varied considerably among the studies. Out of the 26 studies included in this review, 17 had controls with no active intervention. Among these 17, 10 were continued on the usual daily routine, and seven continued standard medical and/or surgical therapy for the underlying condition. Nine other studies used a specific intervention in the control arm. Two studies each used attention control (AC) [29,20], health education [41,18], and physical exercises [19,40] in the CG. Other interventions in the CG included one on using relaxing music [36], one on dietary interventions [38], and another on watching a comedy movie or reading a book [28]. Five studies had waitlisted controls [17,34,33,22,21]. Two studies used a three-arm study, with two arms being the control arms and one being the yoga arm [19,28]. 

Period of intervention: The total duration of yoga administered was quite variable in the studies included. The duration ranged from 20 minutes to 10,080 minutes, with a median IQR of 1640 (990, 3165) minutes. The duration of each session ranged from 12 minutes to 120 minutes, with a median IQR of 60 (30, 87.5) minutes. The frequency of the sessions ranged from once per week to all seven days in the week, i.e., daily sessions, with a median IQR of 3.5 (2, 6) sessions per week. The total number of weeks for interventions that were multi-sessions ranged from seven to 24 weeks, with a median IQR of 12 (8,12).

Region and race: Among the 26 studies included, 12 were performed in the Indian subcontinent, 10 in the United States, and one each in China, Taiwan, Korea, and Thailand. Gender information was provided in 16 of the 26 studies, for a total of 1362 subjects. Regarding ethnicity, only four studies mentioned the race and ethnic characteristics of the participants. Race and ethnicity were available for 426 participants in these four studies [17-20,41]. Among these, whites constituted the majority (367, 86.2%); others included 25 Blacks (5.8%), five African Americans (1.1%), seven Asians (1.6%), and 18 others (4.2%). Multiple studies have been conducted in Asian and South Asian regions, but due to a lack of thorough racial and ethnic profiling in the study, these participants were not blindly categorized as per the region of study and were excluded from race and ethnicity analysis.

Inflammatory Biomarkers and the Impact of Yoga on Inflammation

**Figure 2:**
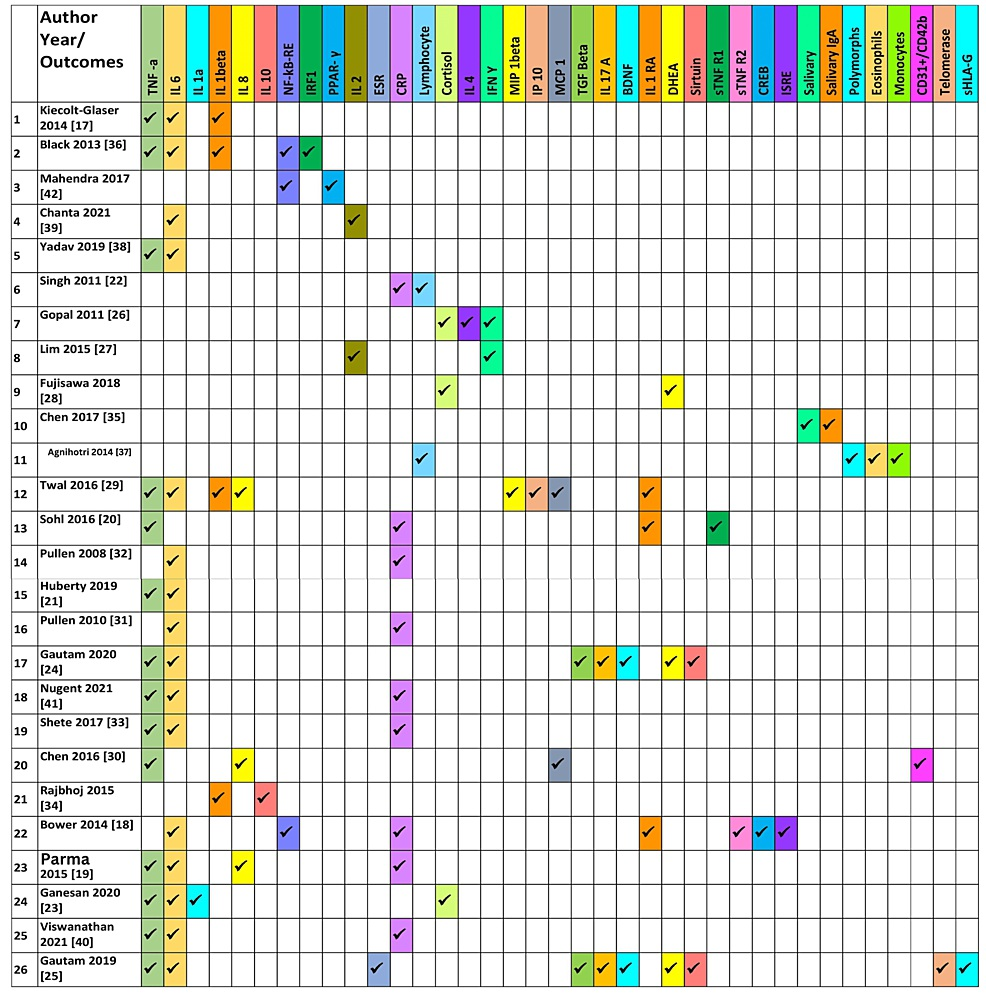
The types of outcomes reported in various studies

**Figure 3:**
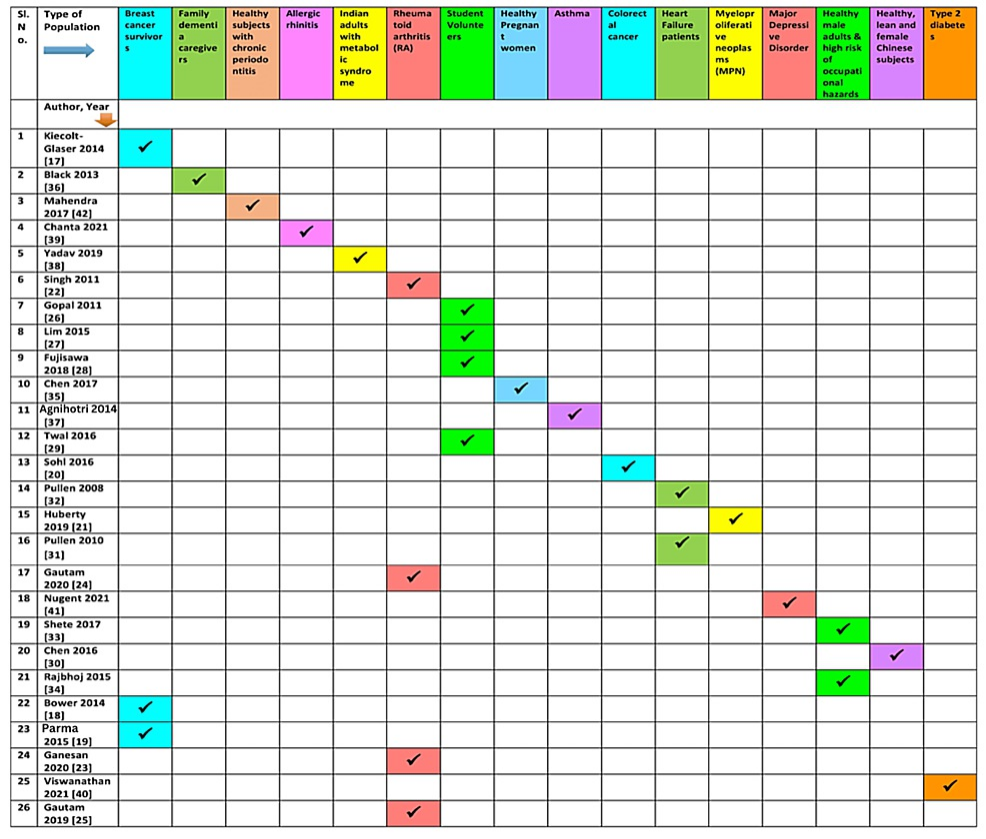
The types of population reported in various studies

**Figure 4:**
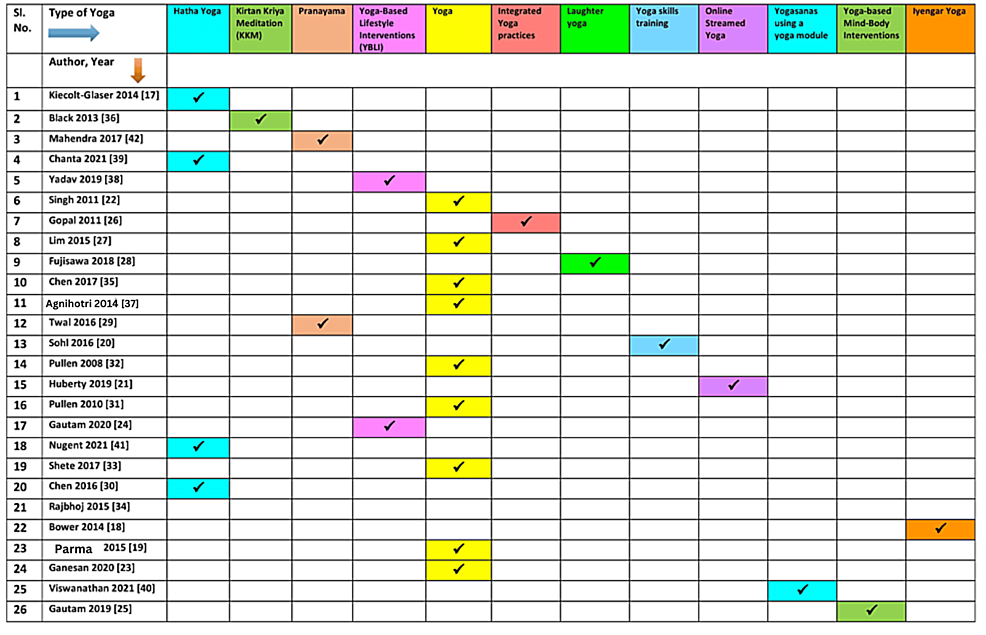
The types of yoga specified as an intervention in the included studies

**Figure 5:**
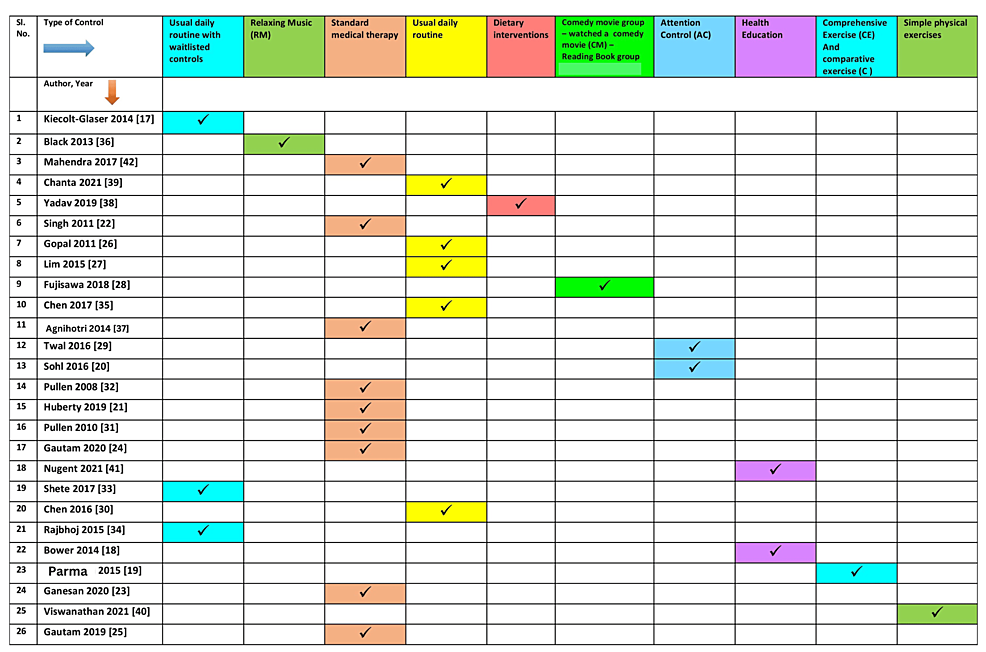
The types of interventions stated as controls in the included studies

Though the studies had heterogeneity in varied aspects, most of them, except for two studies [19,20], reported favorable outcomes with yoga intervention. The most commonly reported inflammatory markers included IL-6 (n=17), TNF-a (n=13), CRP (n=10), and IL-1b (n=5). Those reporting a combination of inflammatory markers, IL-6, and TNF-a combined, were evaluated in 13 of the 26 studies. The underlying medical and surgical conditions studied included cancer (breast and colorectal), rheumatoid arthritis, chronic heart failure, asthma, chronic periodontitis, and allergic rhinitis. Table 2 and Figure 2 show each study as a row with reported outcomes as columns. Additionally, Figure 3, Figure 4, and Figure 5 depict the types of yoga, population characteristics, and control groups, respectively, mentioned in the various studies included in this review.

**Table 2:** The important outcomes, adverse events, and limitations of the studies included in this review YG: Yoga group, CG: Control group, CRP: C-reactive protein, hsCRP: High-sensitivity C-reactive protein, DHEA: Dehydroepiandrosterone, DHEAS: Dehydroepiandrosterone-sulfate, IFN: Interferon,  NF-kB: Nuclear factor kappa B, PPARy: Peroxisome proliferator-activated receptor gamma, TNF: Tumour necrosis factor, TGF: Transforming growth factor, IRF1: Interferon response factor 1, DI: Dietary intervention, PROBE: Prospective randomized open, blinded end-point, LC: Lymphocyte count, TLC: Total lymphocyte count, SSRI: Selective serotonin reuptake inhibitor, EMPs: Endothelial microparticles, ANCOVA: Analysis of covariance

No.	Author and year of publication	No. of cases enrolled in YG, and the no. that completed the trial	No. of participants enrolled in CG, and the no. that completed the trial	Outcomes measured	Timing of outcome assessment	Outcomes regarding inflammatory biomarkers after yoga compared to baseline	Adverse events in YG	Adverse events in CG	Major limitations
1	Kiecolt-Glaser et al., 2014 [17]	100, 96	100, 90	TNF-a, IL-6, IL-1b	Baseline, immediately post-intervention, and at three months	At three months post-treatment, IL-6 (p=0.027), TNF-a (p=0.027), and IL-1b (p=0.037) were significantly lower for YG compared with the CG. Planned secondary analyses at three months post-treatment showed that increasing yoga practice also led to a decrease in IL-6 (p=0.01) and IL-1 (p=0.03) production but not in TNF-a production (p=0.05).	Four recurrent breast cancers (not considered intervention-related). Two events attributable to yoga (recurrence of chronic back and/or shoulder problems)	NI	No active CG. The increased attention or group support may have produced nonspecific treatment benefits. No mention of how the missing data was handled.
2	Black et al., 2013 [36]	25, 23	20, 16	IL-1B, IL-6, NF-kB-response elements, TNF, interferon response factor 1 (IRF1)	Baseline and at eight weeks	KKM resulted in the downregulation of transcripts involved in pro-inflammatory cytokines and activation-related immediate early genes. Reduced NF-kB signaling and increased activity of IRF1 in structuring these effects (both p <0.05). But exact values are not provided.	NI	NI	Missing data was not considered in the analysis. Small sample size.
3	Mahendra et al., 2017 [42]	30, 30	30, 30	NF-kB, peroxisome proliferator-activated receptor gamma (PPARγ)	Baseline and at three months	There was a statistically significant reduction in the expression of NF-kB and an increase in PPARy expression levels in the pranayama group in comparison with the CG (p <0.001). Endogenous PPARy has shown anti-inflammatory effects by causing down-regulation of NF-kB.	NI	NI	Baseline characteristics of the participants are not mentioned. Small sample size.
4	Chanta et al., 2021 [39]	15, 13	15, 14	IL-2, IL-6	Baseline and at eight weeks	The IL-2 levels in the nasal secretion of the YG were significantly higher after training and were also higher than in the CG, but IL-6 showed no difference in both groups. Increased nasal secretion of IL-2 and IL-6 are considered anti-inflammatory for allergic rhinitis.	NI	NI	Baseline characteristics are provided for patients who completed the trial and not for the initially recruited patients. The mean and median values of IL-2 and IL-6 are not provided.
5	Yadav et al., 2019 [38]	130, 89	130, 79	IL-6 (pg/mL), TNF-a (pg/mL)	Baseline, at two weeks and 12 weeks	IL-6 levels significantly decreased in YG, but no significant effect on the levels of TNF-a. In the solely dietary intervention (DI) group, no notable changes were seen.			The number of patients at baseline that are sampled in the baseline and in further time points do not match with those mentioned in the prospective randomized open, blinded end-point (PROBE) design. However, there were higher dropout rates and no mention of how the missing data was accounted for.
6	Singh et al., 2011 [22]	40, 40	40, 40	LC (%), CRP (mg/L)	Baseline and at 40 days	After the pranayama intervention, there was a significant reduction (towards normalcy) in LC, and CRP (i.e., p <0.01, and p <0.01, respectively) among the participants. On the contrary, there was no significant reduction in aforesaid parameters among the controls.	NI	NI	Small sample size. Lack of an active CG. The exact values of statistical analysis are not provided.
7	Gopal et al., 2011 [26]	30, 30	30, 30	Cortisol (ng/ml), IL-4 (mg/mL), IFN-y (pg/mL), IFN-γ/IL-4 ratio	Baseline and at eight weeks	During the examination stress (which was the condition under the study), the increase in serum cortical and decrease in serum IFN-y in the YG was less significant (p <0.01) than in the CG (p <0.001). The decrease in serum IFN-y in the control group was statistically significant (p <0.01). Both the groups demonstrated an increase in serum IL-4 levels, the changes being insignificant for the duration of the study. Yoga resisted impairment of cellular immunity seen in examination stress in this study. The increase in serum cortisol in the CG was higher and more significant than that observed in the study group. The decrease in serum IFN-y indicates a decline in cellular immunity with examination stress.	NI	NI	Small sample size
8	Lim et al., 2015 [27]	12, 12	13, 13	IL-12, IFN-y	Baseline and at 12 weeks	Yoga practice significantly increased immune-related cytokines, such as IL-12, and IFN-Y, in serum (p <0.05 or p=0.01). Yoga partially improved the immune function.	NI	NI	Sample size was small. Lack of a parallel CG. The CG freely accessed their physical exercise activities as well as their social activities during experimental periods, which could have affected the final levels of inflammatory markers.
9	Fujisawa et al., 2018 [28]	40, 40	80, 80	Cortisol (nmol/L), dehydroepiandrosterone (DHEA) (nmol/L0), cortisol/ DHEA ratio	Baseline and immediately post intervention	Laughter yoga decreased cortisol levels and cholesterol/DHEA (C/D) ratios but did not affect DHEA levels. The effect of spontaneous laughter on the cortisol dynamics lasted longer than that of simulated laughter.	No adverse events were reported by any of the subjects	No adverse events were reported by any of the subjects	The subjects are only young adults in their 20s. No long-term positive effects or adverse events were reported.
10	Chen et al., 2017 [35]	50, 48	51, 46	Salivary cortisol, salivary IgA	Baseline and immediately post intervention	The intervention group had lower salivary cortisol (p <0.001) and higher IgA (p <0.001) levels immediately after yoga than the CG. Specifically, the intervention group had significantly higher long-term salivary IgA levels than the CG (p=0.018). Prenatal yoga significantly reduced the stress hormone cortisol and enhanced the immune biomarker IgA during pregnancy.	NI	NI	Did not measure the effects of prenatal yoga on long-term clinical outcomes and other physiological markers of immune and adrenal function. Not completely blinded.
11	Agnihotri et al., 2014 [37]	138, 121	138, 120	TLC, polymorphs, lymphocytes, eosinophils, monocytes	Baseline and at six months	In the YG, there was a significant decrease in TLC and differential leukocyte count in comparison to CG (p <0.0001). There was no significant change found in polymorphs, lymphocytes, and monocytes in group comparison.	NI	NI	Missing data was not considered in the analysis. The parameters change in the CG, which is significant‚ was not mentioned in the analysis.
12	Twal et al., 2016 [29]	10, 10	10, 10	IL-1b (pg/mL), IL-8 (pg/ml), MCP1 (pg/mL),IL-1Ra, IL-6, IL-10 IL-17, IP-10, MIP-1b, TNF-a	Baseline and at 10 minutes and 20 minutes post intervention	The levels of IL-1b, IL-8, and monocyte MCP-1 in saliva were significantly reduced in the yogic breathing (YB) group when compared to the attention control (AC) group. There were no significant differences between YB and AC groups in the salivary levels of IL-1Ra, IL-6, IL-10, IL-17, IP-10, MIP-1b, and TNF-a.	NI	NI	Small sample size. The cytokine levels at baseline were taken after learning the breathing exercise in the YB group as opposed to before learning the technique. Because participants had already learned the technique, the cytokine levels may not represent a true baseline measurement. A single session of 20-minute intervention only.
13	Sohl et al., 2016 [20]	8, 6	7, 5	IL-6, IL-1Ra, sTNF-R1, TNF-a, CRP	Baseline and at eight weeks	No significant changes in inflammatory biomarkers in response to the yoga intervention in patients with colorectal cancer	NI	NI	Small sample size and no long-term follow-up data. Disparity in the amount of intervention received between YG and CG. The median length of each intervention session was significantly different by arm with a median of 30.0 minutes.
14	Pullen et al., 2008 [32]	9, 9	10, 10	IL-6 mg/dL, CRP (mg/dL)	Baseline and at eight weeks	There were statistically significant reductions in serum levels of IL-6 and hsCRP with an eight-week yoga-based program in patients with compensated systolic heart failure (HF) group (all p < 0.005 vs. medical therapy).	NI	NI	Small sample size. Several patients with a skewed distribution between male and female subjects in each group. No active CG.
15	Huberty et al., 2019 [21]	34, 27	28, 21	TNF-a (pg/mL), IL-6	Baseline and at eight weeks	There was a decrease in TNF-a from baseline to week 12 (i.e., 1.3 ± 1.5 pg/ml). Remote blood draw procedures are feasible and the effect size of the intervention on TNF-a was large.	No adverse events	No adverse events	Small sample size and disproportionately more females. Potential confounding factors like the treatment regimens were not taken into account. Long-term outcomes were not considered. Compliance could not be rigorously verified. No healthy CG.
16	Pullen et al., 2010 [31]	21, 18	19, 16	IL-6 mg/dL, hsCRP (mg/dL)	Baseline and at eight weeks	Both IL-6 and hsCRP showed a significant improvement with an eight-week yoga-based program in patients with compensated systolic and diastolic HF. Significant favorable changes in the YG were compared with those in the CG for biomarkers (IL-6, p=0.004; CRP, p=0.016).	NI	NI	Small sample size. Reliance on the exercise log for the documentation of home activities, led to reliability, adherence, and compliance issues. No long-term follow-up. Single hospital setting and only African American population, hence generalizability questionable.
17	Gautam et al., 2020 [24]	33, 31	33, 31	IL-6 (pg/ml), TNF-a (pg/mL), TGF-b (ng/mL), IL-17A (pg/mL)	Baseline and at eight weeks	The YG observed significant improvements in the levels of markers that influence the psycho-neuro-immune axis, i.e., brain-derived neurotrophic factor (BDNF), dehydroepiandrosterone-sulfate (DHEAS), sirtuin, and B-endorphin (all p <0.001). The YG showed downregulation of IL-6, TNF-a, and upregulation of TGF-b (all p <0.05). Yoga practice is associated with a significant reduction in inflammatory cytokines, and the elevation of mind-body communicative markers, in patients with RA.	NI	NI	Small sample size. Lack of an active CG. No balance of sex, more females than males. No follow-up period.
18	Nugent et al., 2021 [41]	48, 48	39, 39	IL-6, TNF-a, CRP	Baseline, and at three weeks and eight weeks	Significant reduction in IL-6 concentrations in the YG relative to the health education CG (p <0.05). Both TNF-a and CRP did not show any significant reductions in the treatment group by mean slope or intercept (p=0.125 and p=0.42, respectively)	no mention	no mention	No long-term follow-up to ascertain whether the improvements were maintained. Small sample size. Whether selective serotonin reuptake inhibitor (SSR)I itself can reduce inflammatory marker levels was not evaluated.
19	Shete et al., 2017 [33]	24, 19	24, 18	IL‑6 (pg/ml), hsCRP (μg/ml) TNF‑α (pg/ml)	Baseline and at 12 weeks	The results within the group comparison revealed significant changes in hsCRP (p <0.01), IL-6 (p <0.001), and TNF-a (p <0.001) in the YG. Comparison between YG and CG revealed significant changes in IL-6 (p < 0.01), TNF-a (p < 0.01), and hsCRP (p < 0.01).	NI		Selection bias, small sample size, lack of adjustment for characteristics. Undefined age range and significant dropout rate Other well-established risk factors such as alcohol intake, parental history of diseases, and physical activity levels were not included in this study.
20	Chen eta al., 2016 [30]	15, 15	15, 15	IL-8 (pg/ml), MCP-1 (pg/mL), TNF-a (pg/mL), CD31+/CD42b, EMPs, stimulated IL-6, stimulated TNF-a, stimulated IL-1b	Baseline and at eight weeks	Reduced secretion of IL-6, TNF-a, and IL-1 levels after yoga training compared to CG. The YG also demonstrated damped cytokines secretion including IL-6, TNF-a, and IL-1 levels (stimulated) compared to the pre-yoga condition and CG. However, statistical significance was not analyzed.	NI	NI	Small sample size and only females. The measurement methods used were not standardized. The mean and median values of various important outcomes like CD31+/ TNF-alpha/IL- 1beta/LPS/IL6 were not provided.
21	Rajbhoj et al., 2015 [34]	24, 19	24, 18	IL-1b, IL -10	Baseline and at 12 weeks	The YG showed a significant decrease in IL-1b and a significant increase in IL-10 (p < 0.05, anti-inflammatory), whereas the CG revealed no change in IL-1b (p >0.05) and IL-10 (p >0.05). The YG also had a significantly lower level of IL-1b and an increase in IL-10 compared to the CG (p <0.05).	NI	NI	Small sample size
22	Bower et al., 2014 [18]	16, 13	15, 15	Soluble TNF receptor type II (sTNFRII) (pg/ml), IL-1R antagonist (pg/ml), IL-6 (pg/ml), CRP (mg/L), salivary cortisol diurnal, slope salivary cortisol, area under the curve, NF-kB, CREB	Baseline, and immediately post intervention and at three months	The YG showed reduced activity of the pro-inflammatory transcription NF-kB, increased activity of the anti-inflammatory glucocorticoid receptor, and reduced activity of CREB family transcription factors relative to controls (all p <0.05). Plasma levels of sTNF-RII remained stable in the YG (a marker of TNF activity), whereas levels of this marker increased in the health education group (p=0.028). A similar nonsignificant trend was observed for the IL-1R antagonist (p=0.16), as well as in CRP, IL-6, and diurnal cortisol measures.	NI	NI	Small, highly selected sample. The yoga and health education conditions were not matched for class frequency or duration. The YG group received 12 hours more of intervention compared to the CG. It is possible that benefits seen in the YG may be attributable in part to the higher number of intervention hours received. No long-term follow-up results. Levels of inflammatory markers were quantified at the pre- and post-intervention assessments only and not at three months post-intervention due to funding constraints.
23	Parma et al., 2015 [19]	31, 20	31 clinically evaluable, 32 completed; 26 clinically evaluable, 26 completed	IL-6 (pg/mL), IL-8 (pg/ml), TNF-a (pg/mL), CRP (ug/mL)	Baseline and at six months	No significant improvements were seen in inflammatory serum markers nor a one-way analysis of covariance (ANCOVA), while controlling for age, BMI, cardiorespiratory capacity, and serum marker baseline values.	NI	NI	Small sample size. High attrition rate. No knowledge of whether outside of studio yoga classes, participants also attended other activities, which could have confounded the results.
24	Ganesan et al., 2020 [23]	83, 68	83, 75	IL-1a (pg/mL), IL-6 (ng/mL), TNF-a (pg/mL), cortisol (ng/mL)	Baseline and at 12 weeks	In both YG and CG, IL-1a, IL-6, TNF-a, and cortisol decreased after 12 weeks, but IL-1a and cortisol decreased more significantly in YG than in CG.	NI	NI	No long-term follow-up data. There was no analysis of the total numbers that were included in the trial. Patients with low-to-severe disease activity were included in the study. Subgroup analysis of low, moderate, and severe disease activity patients was not done because it was underpowered to detect the difference (more patients were needed in each subgroup).
25	Viswanathan et al., 2021 [40]	200, 150	200, 150	IL-6 (pg/mL), TNF-a (pg/mL), hsCRP(ng/mL)	Baseline and at three months	In the YG, the levels of IL-6 (pg/ml), hsCRP, and TNF-a (pg/ml) decreased significantly (p=0.02, p <0.001, and p=0.01, respectively). In the CG, IL-6 (pg/ml) showed significant reduction (p=0.01), and TNF-a (pg/ml) and hsCRP showed insignificant reduction at three months (p=0.35, p=0.67, and p=0.18, respectively).	NI	NI	Lack of long-term follow-up. High dropout rate after recruitment but no mention of the cause of such high dropout rates. The dropouts were excluded from the final analysis.
26	Gautam et al., 2019 [25]	36, 30	36, 32	IL-6 (pg/mL), TNF -a, IL-17A (pg/mL), TGF-b (ng/ml), CRP (ng/ml), ESR (mm/1st hour), telomerase activity (IU/cell), immune-modulatory molecule (sHLA-G)	Baseline and at eight weeks	After eight weeks of yoga-based mind-body intervention, there was a significant decrease in the severity of RA as seen by the reduction in levels of various systemic inflammatory markers.	NI	NI	Small sample size. Lack of an active CG.

Effect of Yoga on Patients With Cancer

Population, intervention, and controls: Five of the 25 studies report the effects of yoga on cancer patients: three with breast cancer [17-19], one with colorectal cancer [20], and one with MPN [21]. All the studies used different methods of yoga intervention, viz., Hatha yoga, Iyengar yoga, online streamed yoga, yoga, and yoga skills training. The controls used were also different among the groups (waitlisted controls, health education, comprehensive and comparative exercise, and attention control).

Outcome: Three of these reported favorable outcomes with the yoga intervention [17,18,21] while two reported no beneficial effects [19,20]. The beneficial outcomes on inflammatory markers were not related to any particular stage of cancer. The various outcomes measured included TNF-a, IL-6, IL-1b, IL-8, CRP, NF-kb, CREB family transcription factors, soluble TNF receptor II (sTNF-RII), IL-R1a, and sTNF-R1.

Kiecolt-Glaser et al. evaluated the effect of Hatha yoga on 186 breast cancer survivors and waitlisted controls who followed their usual daily routine. There was a significant decline in the levels of TNF-a (p=0.027), IL-6 (p= 0.027), and IL-1b (p=0.037) at three months post-yoga compared to the CG. However, there was no active group in the control arm in this study, and the yoga intervention was not supervised [17].

Bower et al. evaluated the effect of Iyengar yoga (a type of Hatha yoga) on breast cancer survivors with cancer-related fatigue with CG receiving health education. Inflammatory markers as well as pro- and anti-inflammatory transcription factors were evaluated. There was a significant reduction in the transcription of the pro-inflammatory NF-kB and CREB family transcription factors (both p <0.05) in the YG compared to the CG and an increase in the activity of the anti-glucocorticoid receptor (p <0.05). In the YG, levels of soluble TNF receptor II (sTNF-RII, a marker of TNF activity) remained stable while they increased in the CG (p=0.028). However, the YG received 720 minutes more intervention compared to the duration of health education in the CG. Moreover, the inflammatory markers were estimated before and immediately after the first yoga setting and not at 12 weeks, apparently due to funding constraints [18].

Huberty et al. (2019) evaluated 48 subjects of MPN; 27 in the YG received online streamed yoga, and 21 in the CG were waitlisted controls and followed their usual daily routine. At 12 weeks, TNF-a decreased compared to baseline (i.e., 1.3 ± 1.5 pg/ml, effect size (ES) = -0.87), as did the levels of IL-6 (ES = -0.26). The sample size was small and without a healthy active control group [21].

Two studies evaluating the effects of yoga on cancer patients found no favorable impact. Parma et al. evaluated 72 breast cancer survivors; 20 received yoga and two control arms, and each group of 26 received comprehensive exercise and comparative exercise as control conditions. At the end of 24 weeks, no significant differences were observed between the YG and CG in the levels of inflammatory markers, IL-6 (p=0.65), IL-8 (p= 0.95), TNF-a (p=0.06), and CRP (p=0.49). The high attrition rate in the intervention arm and the small sample size could have been the reasons behind these inconclusive results [19].

Sohl et al. evaluated 11 subjects who had received chemotherapy with yoga skills training in the YG and attention control in the CG. There was no significant change in the YG compared to the CG in the estimates of inflammatory markers: IL-6 (p=0.715), IL-R1a (p=0.855), sTNF-R1 (p=0.715), TNF-a (p=0.273), and CRP (p=0.361). But the study had a very small number of participants in the YG (n=6) (Tables 1-2) [20].

Effect of Yoga on Patients With Rheumatoid Arthritis

Population, intervention, controls, and outcome: Four studies evaluated the effects of yoga on RA. Two studies used yoga-based lifestyle interventions (YBLI), and two studies used yoga. All the studies used standard medical therapy as an intervention in the CG. The various outcome measures used included IL-6, TNF-a, IL-17A, CRP, ESR, and an increase in the anti-inflammatory marker TGF-b. Gautam et al. evaluated levels of inflammatory markers and mind-body communicative markers in RA patients, with 30 in the YG receiving YBLI and 32 in the CG receiving the usual standard medical therapy for RA. At the end of eight weeks, YG showed a significant reduction in the pro-inflammatory markers IL-6 (p <0.002), TNF-a (p <0.001), IL-17A (p < 0.001), CRP (p=0.016), ESR (p=0.003), and an increase in the anti-inflammatory marker TGF-b (p=0.003). However, the sample size was small, and there was a lack of an active control group [25].

In another such study, Gautam et al. again evaluated the levels of inflammatory markers and mind-body communicative markers in RA patients, with 31 in the YG receiving YBLI and 32 in the CG receiving the usual standard medical therapy for RA. At the end of eight weeks, YG showed a significant reduction in the levels of pro-inflammatory markers IL-6 (p <0.002) and TNF-a (p <0.001), except IL-17A (p <0.001), and an increase in the anti-inflammatory marker TGF-b (p=0.003). Again, the sample size was relatively small, without any active CG, and had more females than males [24].

Ganesan et al. evaluated 68 RA patients in YG who received yoga for 12 weeks and 75 RA patients in CG who received standard medical therapy for RA alone. After 12 weeks, in both YG and CG, IL-1a, IL-6, TNF-a, and cortisol showed down-regulation, but IL-1a and cortisol decreased more significantly in YG than in CG (p=0.017 and p=0.006, respectively) [23].

Singh et al. evaluated 40 RA patients each in the YG and CG who received yoga and standard medical therapy, respectively. After 40 days, there was a significant decrease in lymphocyte count (LC) and CRP (p <0.01 and p <0.01, respectively) in the YG and no significant reduction in the same parameters in the CG. The sample, though, was small, with a lack of an active CG (Tables 1-2) [22].

Effect of Yoga on Patients With Heart Failure

Two studies evaluated the effect of yoga on patients with HF, one with compensated systolic HF patients [32] and the other with both compensated systolic and diastolic HF [31]. In both studies, the CG received standard medical care for HF. Both studies combined had 27 in the YG and 26 in the CG. At the end of eight weeks, both studies showed significant reductions in the levels of IL-6 and high-sensitivity (hs) CRP (all p <0.05). However, both studies had very small sample sizes, an imbalance in baseline characteristics, and compliance issues, and featured only the African American population.

Effect of Yoga on Healthy Volunteers

Seven studies evaluated the effect of yoga on healthy subjects. Four of these studied healthy student volunteers exposed to varied lifestyle stressors [26-29], one each studied healthy pregnant females [35] and healthy lean females [30], and one observed family dementia caregivers [36]. The outcome measures and the control condition in the studies enrolling healthy volunteers varied among the studies. While in four studies [26,27,35,30] the CG followed the usual daily routine, one study employed two control arms, with comedy movie watching in one and reading a book in another [28]. The other studies used attention control [29] and relaxing music (RM) [36] as interventions in the control arm.

Gopal et al. exposed student volunteers to academic exam stress, and at the end of eight weeks, there was a less significant increase in serum cortisol and reduction in IFN-g levels in the YG compared to the CG (p <0.01), suggesting yoga inhibited stress-related excessive cytokine release [26]. In the study by Lim et al., 12 weeks of yoga enhanced IL-12 (p < 0.05) and IFN-g (p = 0.01), suggesting partial improvement in immune functionality [27]. Fujisawa et al. employed laughter yoga in the YG and found that after 30 minutes of intervention, the YG significantly reduced cortisol levels (p=0.016) and the cortisol/dehydroepiandrosterone (DHEA) ratio (p=0.001) but did not affect DHEA levels alone (p p=0.517) [28]. Twal et al. also showed significant down-regulation of IL-1b, IL-8, and monocyte MCP-1 in saliva, which were significantly reduced in YG compared to CG [29]. A major drawback of both the Fujisawa et al. and Twal et al. studies was the assessment of inflammatory outcomes immediately after the 30- and 20-minute interventions, respectively.

Two studies evaluated the effects of yoga on healthy Chinese females [30,35]. Chen et al. evaluated 94 healthy pregnant females. Immediately post-intervention, the YG had significantly lower salivary cortisol (p<0.001) and higher IgA levels (p<0.001) compared to the CG. After 20 weeks, YG had significantly higher IgA levels than CG [35]. Chen et al. evaluated 37 healthy lean Chinese females and found down-regulation of inflammatory markers IL-6, TNF-a, and IL-1 in the YG compared to the CG, but statistical significance was not provided. The secretion of stimulated cytokines showed a significant decline in the YG compared to the CG (stimulated IL-6, p<0.05; TNF-a, p<0.05; and IL-1a, p<0.05) [30].

Black et al. evaluated 39 subjects who are involved in providing care to dementia patients. Twenty-three subjects in the YG who received KKM showed downregulation of pro-inflammatory activation-related immediate early genes, i.e., reduced expression of NF-kB and increased activity of IRF-1 (both p <0.05) compared to the CG, which received RM. The sample size was small, and intention-to-treat (ITT) analysis was not done for the missing data (Tables 1-2) [36].

Effect of Yoga on a Healthy Population Exposed to Occupational Hazards

Two studies [33,34] evaluated the effects of yoga on healthy subjects with a high risk of occupational hazards employed in industries manufacturing steel, chemicals, and paints [33,34]. Both studies employed waitlisted controls who followed the usual daily routine. Both studies employed 37 patients each and 12 weeks of yoga intervention. At the end of 12 weeks, both studies reported a significant decline in the inflammatory markers within the YG (IL-6 (p <0.001), hsCRP (p <0.01), and TNF-a (p <0.001) in the study by Shete et al. [33], and IL-1b (p<0.05) in Rajbhoj et al. [34]) as well as in comparison to the CG (Shete et al.: IL-6 (p <0.01), hsCRP (p <0.01), and TNF-a (p <0.01) [33]; Rajbhoj et al.: IL-1b (p <0.05) [34]). In addition, Rajbhoj et al. reported an increase in the anti-inflammatory cytokine IL-10 both within YG and in comparison to CG (p <0.05) [34]. Both studies had small sample sizes, selection bias, and significant dropouts.

Effect of Yoga on Other Miscellaneous Chronic Medical Conditions

Mahendra et al. evaluated 60 patients with chronic periodontitis, and those in YG had a statistically significant reduction in the expression of NF-kB and an increase in PPARy expression levels (anti-inflammation causes down-regulation of NF-kB) compared to the CG (received standard of care) (p <0.001) [42]. Chanta et al. evaluated 27 subjects with allergic rhinitis, and those in the YG had higher nasal secretion of IL-2 levels compared to those in the CG (who followed the usual routine), but IL-6 showed no difference in both groups. In this study, increased nasal secretion of IL-2 and IL-6 was considered anti-inflammatory for allergic rhinitis [39]. Yadav et al. evaluated 168 Indian adults with metabolic syndrome, and those in the YG showed a significant decline in IL-6 levels at the end of 12 weeks (p <0.001). No significant changes were seen in the CG (received dietary interventions). No between-group comparison was done in this study [38]. 

Kant et al. evaluated 240 patients with asthma and those in the YG and found a significant decrease in the TLC and differential leukocyte count (DLC) compared to the CG (received standard of care) at the end of 24 weeks. No significant change was noticed in the levels of polymorphs, lymphocytes, and monocytes [37]. Nugent et al. evaluated 87 patients with major depressive disorder, and those in the YG had a significant reduction in the IL-6 levels (p <0.05) compared to the CG (received health education). No significant changes were seen in the TNF-a (p=0.125) and CRP levels (p=0.42) [41].

Vishwanath et al. evaluated 300 patients with type 2 diabetes mellitus (DM), and the levels of IL-6 (p=0.02), hsCRP (p <0.001), and TNF-a (p=0.01) in the YG showed a significant reduction at 12 weeks compared to baseline. In the CG (received simple physical exercises), IL-6 showed a significant reduction (p=0.01), while hsCRP and TNF-a did not show any significant reduction. No between-group comparison values were provided (Tables 1-2) [40]. Two studies [37,22] mentioned findings on blood counts, an indirect marker of inflammation. Both studies found a significant decrease in TLC in the YG pre- and post-intervention but no significant reduction compared to CG (Table 2) [37,22].

Meta-Analysis of Commonly Reported Markers

**Figure 6:**
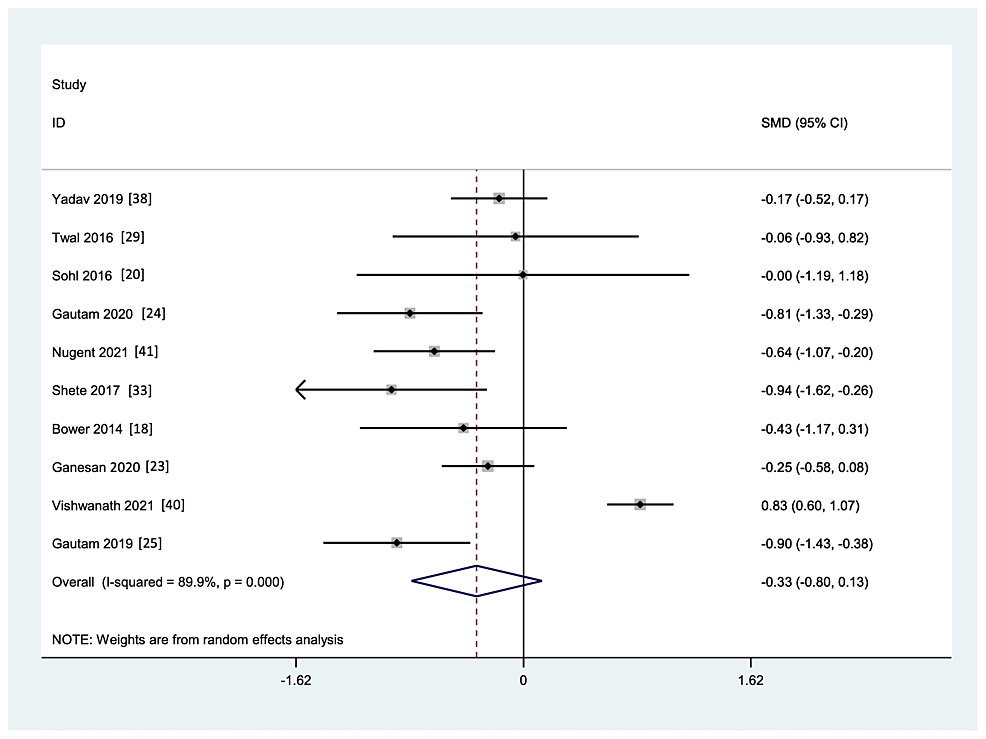
Forest plot showing the effect of yoga on inflammatory markers IL-6 versus control interventions, standard medical therapy, or the usual daily routine

**Figure 7:**
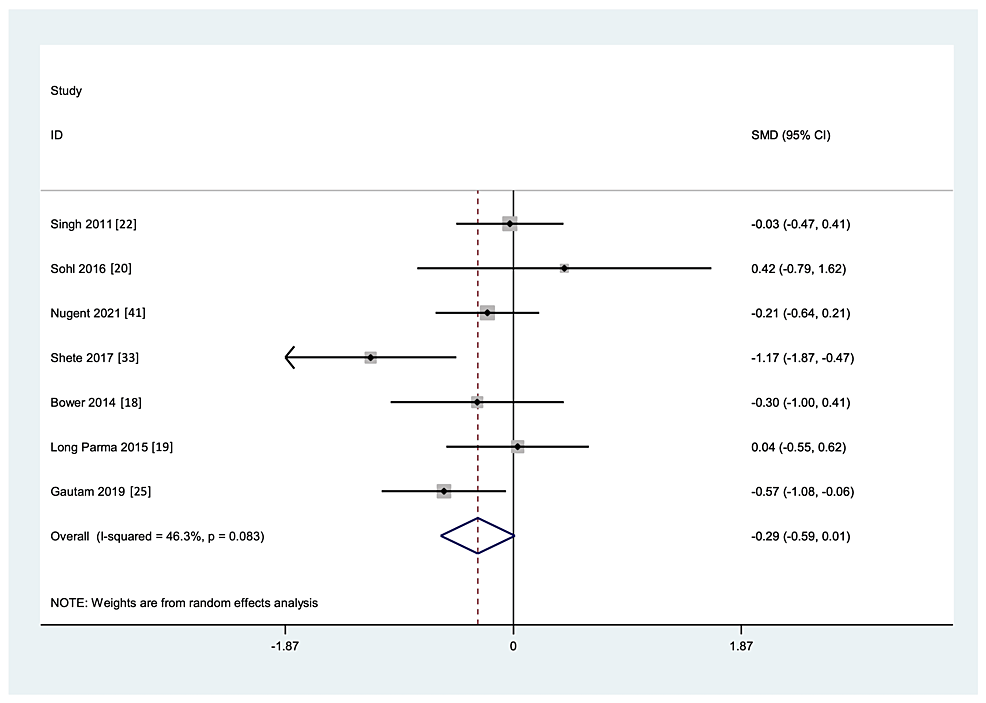
Forest plot showing the effect of yoga on inflammatory marker TNF-alpha versus control interventions, standard medical therapy, or usual daily routine.

**Figure 8:**
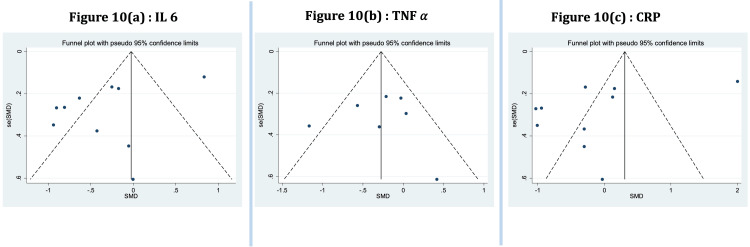
Funnel plot and Egger’s regression test for publication bias (a): Studies reporting IL-6; (b): Studies reporting TNF-a; (c): Studies reporting CRP TNF: Tumour necrosis factor, CRP: C-reactive protein

Markers reported in at least four studies in which data was available for both the YG and CG pre- and post-intervention were included for quantitative analysis. These were IL-6, TNF-a, and CRP. As shown in Figure 6 and Figure 7, yoga intervention showed a favorable effect on the levels of IL-6 and TNF-a markers, respectively, but it was not statistically significant, and there was considerable heterogeneity and publication bias among the studies included (p <0.001, and I² = 89.9% and 95.9%, respectively, for IL-6 and TNF-a). A funnel plot shows the publication bias (Figure 8).

**Figure 9:**
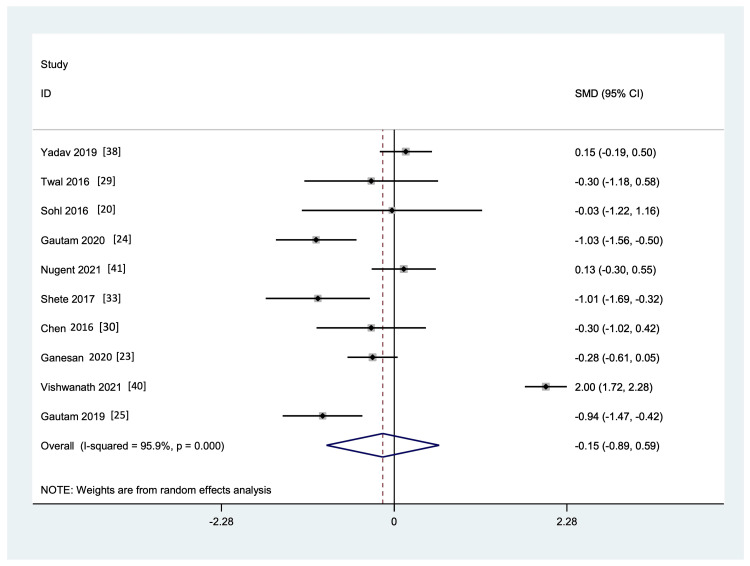
Forest plot showing the effect of yoga on inflammatory marker CRP versus control interventions, standard medical therapy, or usual daily routine

For CRP, the included studies showed a non-significant favorable beneficial effect with yoga, but there was moderate heterogeneity amongst the included studies with publication bias (p <0.083). Figure 9 shows the effect and heterogeneity (I^2^ =46.3%), and Figure 8c shows the publication bias.

Discussion

This review included 26 studies and 2091 participants, with considerable heterogeneity in the type of yoga used, the duration of the intervention provided, the type of control group used, and the outcome measures. Nonetheless, most of the studies, barring two, found a favorable outcome of yoga on inflammatory cytokines and various other biomarkers. These findings are concordant with previous reviews on this subject [8,12,43-45]. The 26 studies included in this review reported on diversified medical and surgical conditions and also evaluated healthy subjects. Five of the 26 studies reported on cancer, four on RA, two each on HF, and subjects exposed to the risk of occupational hazards. Interventions in the form of yoga resulted in a favorable outcome in the levels of inflammatory markers in three of the five studies involving cancer subjects, all the studies on rheumatoid arthritis, HF, subjects exposed to occupational hazards, healthy subjects, and miscellaneous chronic conditions ranging from diabetics to asthmatics.

Yoga therapies may also have an anti-inflammatory effect through their effects on the sympathetic and parasympathetic nervous systems. Apart from yoga postures, which were the most emphasized ones in the studies, 17 studies employed pranayama, or yogic breathing, and 11 studies employed relaxation techniques. Pranayama is the yogic art of breathing, comprising cycles of deliberately slow and fast breathing. Previous studies have shown a beneficial effect of pranayama on autonomic nervous system functioning [46]. Pranayama has been shown to increase parasympathetic activation and damp sympathetic reactions, favorably shifting the balance [47], especially the slow breathing shifts [48]. Therefore, moderate movements and specific breathing exercises from yoga may help regulate the relaxation of the autonomic nervous system.

Another crucial finding in this study is that a varied list of different inflammatory biomarkers was used by the studies as outcome indicators. As many as 22 inflammatory markers have been reported in the included studies as outcome measures. The use of such a large repertoire of markers makes results heterogeneous, as was faced during this review. The most commonly reported inflammatory markers included IL-6 (n=18), TNF-a (n=13), CRP ( n=9) and IL-1b (n=5). Only those outcomes reported in more than four studies were included in the meta-analysis. In the future, researchers should try to include a minimum conglomerate of commonly reported outcomes like IL-6, TNF-a, and CRP, apart from new and innovative measures, so that the results can be homogenous and comparable, at least in some common measures.

Similarly, regarding the type of intervention, a core set of yoga subcomponents would be desirable, apart from any other newer intervention the authors may contemplate evaluating. Moreover, the duration of the yoga intervention had quite an extended range, lasting from as low as 20 minutes to as high as 10,080 minutes. A short duration of yoga might not be adequate to induce consistent effects on immune markers, and no studies have reported on long-term outcomes. Future researchers should try to incorporate a core set of subcomponents of yoga intervention and an acceptable duration of intervention.

This systematic review gives insights into the evidence for the effect of yoga in the current scenario. There was considerable heterogeneity in the subcomponents of yoga intervention, duration, and outcome measurements. The IL-6 was the most commonly reported outcome in all the studies (n=17). Out of 17 studies, 10 showed a significant reduction in levels of IL-6 (three studies within the group, the other eight studies between YG and CG), three showed no reduction [19,20,29], one showed a significant increase in mucosal secretion (considered protective) [39], and the other three studies showed a non-significant reduction compared to CG [21,18,23]. This inconsistent evidence was probably due to the inherent methodological weakness of the studies. For instance, Twal et al. [29] used only 20 minutes of yoga, and Parma et al. [19] had a high attrition rate, not analyzed by ITT. The effect sizes ranged from small to medium; however, Pullen et al. [31] and Gautam et al. [24] reported relatively large effect sizes (d=1.796 and 0.128, respectively).

Results were more mixed for TNF-a, which was reported in 13 studies. Out of 13 studies, seven showed significant downregulation in the levels of TNF-a, four showed no downregulation [19,20,29,41] and three showed a non-significant reduction [18,21,23] compared to CG. The effect sizes ranged from small to medium; however, Bower et al. [18] and Huberty et al. [21] reported relatively large effect sizes (d=0.892 and -0.87, respectively). Therefore, further studies are needed.

A similarly mixed picture was also noticed in CRP levels. Out of 10 studies reporting CRP, five found a significant reduction in the levels of CRP, two showed a non-significant reduction [18,21], and three showed no reduction [20,29,41] compared to CG. The effect sizes ranged from small to medium; however, Pullen et al. [31] and Huberty et al. [21] reported relatively large effect sizes (d=0.892 and -0.87, respectively). All five studies reporting on IL-1b showed a significant improvement compared to CG [17,29,30,34,36]. All of these markers play crucial roles in the inflammatory response and are therefore essential for a healthy immune system. However, a prolonged rise in any of these indicators or their dysregulation can have negative effects on the immune system. The marker IL-6 has been connected to numerous inflammatory conditions ranging from RA to cancer [49], as have TNF-a [50], CRP [51], and IL-1b [52].

Two studies showed the beneficial effects of yoga on regulating the transcription of genes responsible for downstream pro-inflammatory (NF-kB and CREB) and anti-inflammatory effects (PPARy) [42,18]. Thus, modulating the genetic pathways may be another one of the mechanisms by which yoga can affect inflammation. A previous review by Buric et al., involving 18 studies, had also shown a similar beneficial effect of mind-body interventions on NF-kB expression [53]. Further evidence is needed to unravel the molecular signature of yoga in the inflammatory process.

Three studies [24,25,29] showed a significant reduction in IL-17A, but further studies are needed. Serum cortisol is considered an acute-phase reactant. Three studies [23,26,28] showed a significant reduction in serum cortisol levels, suggesting that yoga interventions may dampen the overactivity of the immune system following stress. However, Fujisawa et al. used only 30 minutes of intervention; hence, further, more rigorous studies are needed [28]. 

Yoga may also benefit the mediation of cellular and adaptive immunity. Two studies [27,26] showed significant upregulation of IFN-g with yoga compared to CG. The IFN-g is a core regulator of cellular immunity, also possessing immune-regulatory, anti-viral, and anti-tumor attributes [54]. In addition, Lim et al. showed significant upregulation of IL-12, and Gopal et al. showed the same for IL-4, both of which are known to stimulate IFN-g. However, larger studies are needed to establish this conclusively.

Two studies showed the enhancement of mucosal immunity by increasing the secretion of protective immune markers. Chanta et al. [39] showed a significant increase in the nasal secretion of IL-2 and IL-6 in allergic rhinitis patients, while Chen et al. [35] showed a significantly higher secretion of salivary IgA in pregnant women, suggesting that yoga has helped fight local pathogen invasion and reduced salivary secretion of cortisol. However, larger studies with an active control group are needed to validate these beneficial effects.

Regarding IL-10, an anti-inflammatory marker, one study by Rajbhoj et al. showed a significant increase [34], while another study by Twal et al. [29] showed no change. However, both studies had very small sample sizes. Regarding another such marker, TGF-b, a significant benefit was shown in two studies [24,25]. However, the patient population was limited to RA patients only, and therefore it needs to be evaluated in future studies in other patient populations as well.

One study showed a significant increase in the expression of the immune modulatory molecule (sHLA-G) [25], and another study showed a significant decline in CTLA4 [24]. Though these two markers play a crucial role in immune modulation in RA patients, both studies had a small sample size and no active control group. Hence, larger studies are needed in the future. Two studies mentioned findings on blood counts, an indirect marker of inflammation. Both studies found a significant decrease in TLC in the YG pre- and post-intervention but no significant reduction compared to the CG [22,37]. 

Assessment of the quality of the studies showed that overall, most of the studies had a high RoB (n=21), three had some concerns [23,25,28] and two studies [24,41] had a low RoB. These findings are in accordance with the previous systematic review by Yeun et al. [43] (eight of 11 trials had a high RoB), but differ from another systematic review by Falkenberg et al. [12], where all the 15 included studies had unclear RoB. In the review by Morgan et al. [8], out of 18 studies, four were high risk, five had moderate risk, and nine had a low RoB. In the review by Djalilova et al. [45], seven of the 15 studies were of high quality, six were average, and two were fair. Khosravi et al. [44] did not perform a RoB quality assessment in their review.

Apart from the poor quality of studies, most investigators did not take into consideration proper sample size calculations for obtaining adequate power for the studies. There was a higher proportion of female participants (61.1% overall), and race/ethnicity data was provided for only four studies. This hinders the generalizability of results.

The current systematic review is unique in various aspects. First, compared to the previous review, the repertoire of immune markers used was quite extended (TNF-a, IL-1b, IL-10, IL-17A, CTLA-4, TGF-b, IL-2, mucosal IgA, cortisol, NF-kB, and PPARy). Previous reviews tended to remove markers that were reported in three or fewer studies. But the present review made no such exclusions. Second, the review evaluated yoga apart from other mind-body therapies. Third, both pro- and anti-inflammatory markers were reviewed, as were the genetic modulators of inflammation. Both healthy populations and subjects with medical conditions were examined, which enhances the generalizability of the results and can suggest the population group in which yoga may have potential benefits.

Limitations 

Considering the heterogeneity in the included studies, the results of this systematic review should be interpreted with caution. Very few studies have used uniform outcome measures, leading to difficulty in pooling data and effective analysis. Also, there were disproportionately more female participants, and the racial and ethnic data of the analyzed population was sketchy. All these hinder the generalizability of the findings. Most of the studies had small sample sizes, resulting in difficulty in obtaining adequate power for the studies. Moreover, most of the studies have reported on statistical significance, and very few studies have provided effect sizes (n=4).

## Conclusions

This systematic review of 26 RCTs revealed that practicing yoga can boost immune function in both patients with various medical conditions and healthy adults. Current evidence suggests yoga may positively impact various immune-mediated and inflammatory diseases by downregulating pro-inflammatory markers and increasing anti-inflammatory markers. The potential impact on the genetic factors of inflammation needs further study. A combination of postures with slow breathing, relaxation techniques, and sustained doses of yoga therapy may be particularly beneficial in reducing chronic inflammation. However, the quality of the studies included was poor, and there was also considerable heterogeneity in the included studies. The correlation of favorable impacts on inflammatory markers with clinically meaningful outcomes also needs to be explored in future studies.

## Figures and Tables

**Figure 10:**
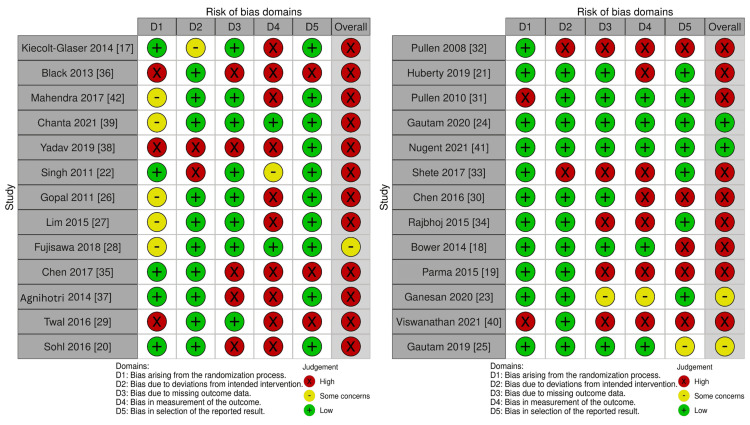
Traffic light plot made using the Cochrane RoB2 tool for RCTs included in this review RoB: Risk of bias, RCTs: Randomized controlled trials

**Figure 11:**
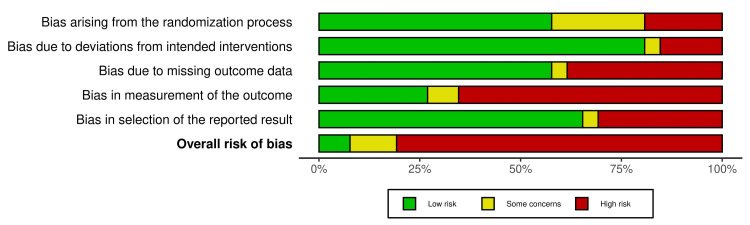
Summary plot of the Cochrane RoB2 tool assessment for RCTs included in this review RoB: Risk of bias, RCTs: Randomized controlled trials

## Appendices

Appendix A

Search Terms

The following search terms were used for the Pubmed database: ((((((((((((((((((((((((((("Yoga" [MeSH Terms] OR "Yoga" [Title/Abstract]) OR "Yogic meditation" [MeSH Terms] OR "Yogic meditation" [Title/Abstract]) OR "Yogic breathing" [MeSH Terms] OR "Yogic breathing" [Title/Abstract]) OR "Yogic postures" [MeSH Terms] OR "Yogic postures" [Title/Abstract])) AND (("Cytokines" [MeSH Terms] OR "Cytokines" [Title/Abstract]) OR "IL-6" [MeSH Terms] OR "IL-6" [Title/Abstract]) OR "IL-1" [MeSH Terms] OR "IL-1" [Title/Abstract]) OR "TNF" [MeSH Terms] OR "TNF" [Title/Abstract]) OR "TNF-alpha" [MeSH Terms] OR "TNF-alpha" [Title/Abstract]) OR "IL-1beta" [MeSH Terms] OR "IL-1beta" [Title/Abstract]) OR "IL-10" [MeSH Terms] OR "IL-10" [Title/Abstract]) OR "IL-12" [MeSH Terms] OR "IL-12" [Title/Abstract]) OR "IFN gamma" [MeSH Terms] OR "IFN gamma" [Title/Abstract]) OR "circulating endothelial microparticles (EMPs)" [MeSH Terms] OR "circulating endothelial microparticles (EMPs)" [Title/Abstract]) OR "C Reactive protein (CRP)" [MeSH Terms] OR "C Reactive protein (CRP)" [Title/Abstract]) OR "Total leucocyte count" [MeSH Terms] OR "Total leucocyte count" [Title/Abstract]) OR "Eosinophils" [MeSH Terms] OR "Eosinophils" [Title/Abstract]) OR "Polymorphs" [MeSH Terms] OR "Polymorphs" [Title/Abstract]) OR "Lymphocytes" [MeSH Terms] OR "Lymphocytes" [Title/Abstract]) OR "IFN-gamma" [MeSH Terms] OR "IFN-gamma" [Title/Abstract]) OR "NK-cytotoxicity" [MeSH Terms] OR "NK-cytotoxicity" [Title/Abstract]) OR "CD4" [MeSH Terms] OR "CD4" [Title/Abstract]) OR "CD8" [MeSH Terms] OR "CD8" [Title/Abstract]) OR "NK cells" [MeSH Terms] OR "NK cells" [Title/Abstract]) OR "Immunoglobulins" [MeSH Terms] OR "Immunoglobulins" [Title/Abstract]) OR "IgA" [MeSH Terms] OR "IgA" [Title/Abstract]) OR "Pro-inflammatory transcription factors NF-kappa B" [MeSH Terms] OR "Pro-inflammatory transcription factors NF-kappa B" [Title/Abstract]) OR "CREB family transcription factors" [MeSH Terms] OR "CREB family transcription factors" [Title/Abstract])

Appendix B

Risk of Bias Assessment

The risk of bias was assessed independently by two assessors, BM and AA, using the Revised Cochrane risk-of-bias tool for randomized trials (Table 3, Figures 10-11) [15].

**Table 3:** Risk of bias assessment of the included studies Y: Yes; N: No; PY: Probably yes; PN: Probably no; NI: No information Domain 1 1.1: Allocation sequence is random; 1.2: Allocation sequence is concealed; 1.3: Baseline differences between intervention groups suggest a problem Domain 2 2.1: Participants are aware of their assigned intervention during the trial; 2.2: Carers and people are aware of participants' assigned intervention during the trial; 2.3: If Y/PY/NI to 2.1 or 2.2, were there deviations from the intended intervention? 2.4: If Y/PY to 2.3, were these deviations likely to have affected the outcome? 2.5: If Y/PY/NI to 2.4, were these deviations from the intended intervention balanced between groups? 2.6: Appropriate analysis was used to estimate the effect of assignment to intervention; 2.7: If N/PN/NI to 2.6, was there potential for a substantial impact (on the result)? Domain 3 3.1: Data for outcome available for all participants randomized; 3.2 If N/PN/NI to 3.1, was there evidence that the result was not biased by missing outcome data? 3.3: If N/PN to 3.2, could missingness in outcome depend on its true value? 3.4: If Y/PY/NI to 3.3, is it likely that missingness in the outcome depended on its true value? Domain 4 4.1: The method of measuring the outcome is inappropriate; 4.2: Measurement or ascertainment of the outcome have differed between intervention groups; 4.3: If N/PN/NI to 4.1 and 4.2, were the outcome assessors aware of the intervention received by study participants? 4.4 If Y/PY/NI to 4.3, was the assessment of the outcome influenced by knowledge of intervention received? 4.5: If Y/PY/NI to 4.4, was the assessment of the outcome influenced by knowledge of intervention received? Domain 5 5.1: Data analyzed per a pre-specified analysis plan; Numerical result being assessed likely to have been selected from 5.2: multiple eligible outcome measurements within the outcome domain, 5.3: multiple eligible analyses of the data

No.	Author and year of publication	Domain 1: Randomization process				Domain 2: Deviations from intended interventions								Domain 3: Missing outcome data					Domain 4: Measurement of the outcome						Domain 5.: Selection of the reported result				Domain 6.: Overall bias
		1.1	1.2	1.3	1.0 Assessor's judgement	2.1	2.2	2.3	2.4	2.5	2.6	2.7	2.0 Assessor's judgement	3.1	3.2	3.3	3.4	3.0 Assessor's judgement	4.1	4.2	4.3	4.4	4.5	4.0 Assessor's judgement	5.1	5.2	5.3	5.0 Assessor's judgement	Assessor's overall judgement
1	Kiecolt-Glaser et al., 2014 [17]	Y	NI	N	Low	Y	Y	Y	Y	Y	Y	NA	Some concerns	N	Y	PN	NA	Low	N	N	Y	Y	Y	High	Y	N	N	Low	High
2	Black et al., 2013 [36]	Y	NI	Y	High	NI	NI	PN	NI	NI	PY	PN	Low	N	N	NI	NI	High	N	N	NI	NI	NI	High	PY	PY	Y	High	High
3	Mahendra et al., 2017 [42]	Y	NI	NI	Some concerns	NI	NI	PN	NA	NA	Y	N	Low	Y	NA	NA	NA	Low	N	N	NI	NI	NI	High	Y	N	N	Low	High
4	Chanta et al., 2021 [39]	NI	NI	N	Some concerns	NI	NI	N	N	NA	Y	NA	Low	N	Y	N	NA	Low	N	N	N	N	NA	Low	Y	N	N	Low	High
5	Yadav et al., 2019 [38]	Y	N	N	High	Y	Y	N	NA	NA	N	Y	High	N	PN	Y	Y	High	N	N	Y	Y	Y	High	Y	N	N	Low	High
6	Singh et al., 2011 [22]	NI	NI	N	Low	NI	NI	N	NA	NA	NI	Y	High	Y	NA	NA	NA	Low	N	N	NI	NI	PN	Some concerns	Y	N	N	Low	High
7	Gopal et al., 2011 [26]	NI	NI	N	Some concerns	NI	NI	N	NA	NA	Y	NA	Low	Y	NA	NA	NA	Low	N	N	NI	NI	NI	High	Y	N	N	Low	High
8	Lim et al., 2015 [27]	NI	NI	N	Some concerns	NI	NI	N	NA	Y	NA	NA	Low	Y	NA	NA	NA	Low	N	N	NI	PN	PN	High	PY	N	N	Low	High
9	Fujisawa et al., 2018 [28]	Y	N	N	Some concerns	Y	Y	N	NA	Y	NA	NA	Low	Y	NA	NA	NA	Low	N	N	Y	PN	PN	Low	Y	N	N	Low	Some Concerns
10	Chen et al., 2017 [35]	Y	NI	N	Low	NI	NI	N	NA	Y	NA	NA	Low	N	PN	Y	Y	High	N	N	NI	PY	PY	High	Y	Y	Y	High	High
11	Agnihotri et al., 2014 [37]	Y	NI	N	Low	NI	NI	N	NA	Y	NA	NA	Low	N	PY	Y	Y	High	N	N	NI	NI	NI	High	Y	N	N	Low	High
12	Twal et al., 2016 [29]	NI	NI	PY	High	NI	NI	N	NA	NA	Y	NA	Low	Y	NA	NA	NA	Low	Y	N	NI	NI	NI	High	Y	Y	Y	High	High
13	Sohl et al., 2016 [20]	Y	Y	N	Low	PY	PY	N	NA	NA	Y	NA	Low	N	PN	PY	PY	High	N	N	PY	PY	PY	High	Y	N	N	Low	High
14	Pullen et al., 2008 [32]	NI	NI	N	Low	NI	NI	PY	PY	PN	Y	NA	High	N	PN	PY	PY	High	PN	N	PY	PY	PY	High	Y	Y	Y	High	High
15	Huberty et al., 2019 [21]	PY	PY	N	Low	Y	Y	N	NA	NA	Y	NA	Low	N	PY	NA	NA	Low	N	N	PY	PY	PY	High	Y	N	N	Low	High
16	Pullen et al., 2010 [31]	N	N	Y	High	NI	NI	N	NA	NA	Y	NA	Low	N	PN	PN	NA	Low	PN	N	N	NA	NA	Low	Y	N	N	Low	High
17	Gautam et al., 2020 [24]	Y	Y	N	Low	Y	Y	N	NA	NA	Y	NA	Low	N	Y	NA	NA	Low	N	N	N	NA	NA	Low	Y	N	N	Low	Low
18	Nugent et al., 2021 [41]	Y	Y	N	Low	NI	NI	N	NA	NA	Y	NA	Low	N	PY	NA	NA	Low	N	N	N	NA	NA	Low	Y	N	N	Low	Low
19	Shete et al., 2017 [33]	Y	NI	N	Low	NI	NI	N	NA	NA	PN	PY	High	N	PN	PY	PY	High	N	N	NI	PY	PY	High	Y	N	N	Low	High
20	Chen et al., 2016 [30]	Y	Y	N	Low	Y	N	N	NA	NA	Y	NA	Low	Y	NA	NA	NA	Low	N	N	NI	NI	NI	High	P	Y	Y	High	High
21	Rajbhoj et al., 2015 [34]	NI	NI	N	Low	NI	NI	N	NA	NA	Y	NA	Low	N	PN	PY	PY	High	N	N	NI	NI	NI	High	Y	N	N	Low	High
22	Bower et al., 2014 [18]	Y	Y	N	Low	N	Y	N	NA	NA	Y	NA	Low	Y	NA	NA	NA	Low	N	N	N	NA	NA	Low	Y	Y	Y	High	High
23	Parma et al., 2015 [19]	NI	NI	N	Low	NI	NI	N	NA	NA	Y	NA	Low	N	PY	PY	PY	High	N	N	NI	NI	NI	High	Y	Y	Y	High	High
24	Ganesan et al., 2020 [23]	Y	Y	N	Low	NI	NI	N	NA	NA	Y	NA	Low	N	PN	PN	PN	Some Concerns	N	N	NI	NI	PN	Some Concerns	Y	N	N	Low	Some Concerns
25	Viswanathan et al., 2021 [40]	Y	NI	Y	High	NI	NI	N	NA	NA	Y	NA	Low	N	Y	Y	Y	High	N	N	NI	NI	NI	High	Y	PY	PY	High	High
26	Gautam et al., 2019 [25]	Y	Y	N	Low	NI	NI	N	NA	NA	Y	NA	Low	N	PY	NA	NA	Low	N	N	N	NA	NA	Low	PN	PY	PY	Some Concerns	Some Concerns

Regarding domain 1, the RoB arising from the randomization process, out of 26 studies, 17 mentioned random sequence generation; only seven had concealed allocation during the entire study, but the majority (n=26) did not have a baseline difference between YG and CG. In summary, most of the studies (n=15) had a low RoB in domain 1; six studies had some concerns, and the remaining six had a high RoB (Figure 11). As for domain 2, the RoB was due to deviations from the intended interventions, most of the studies had a low RoB (21 out of 26), one had some concerns, and four had a high RoB (Figure 11). Domain 3 deals with the RoB due to missing outcome data; out of 26 studies, 18 had missing outcome data. In summary, 14 studies qualified as low-risk in this domain; one had some concerns, and the remaining 11 had a high RoB. Only one study used ITT analysis to account for the missing data. The rest of the studies excluded the missing data from the final analysis and performed a naïve per protocol analysis. (Figure 11). Domain 4 focuses on the RoB in the measurement of the outcome. Most of the studies did not report on double blinding (14 had no information on this). In six studies, the outcome assessor was blinded to the intervention received, and in the remaining six, the outcome assessor was not blinded. Accordingly, most of the included studies (n=16) had a high RoB, seven had a low RoB, and two had some concerns in this regard (Figure 11). Domain 5 highlights the RoB in the selection of the reported result. Most of the studies had a low RoB (n=17), eight had a high RoB, and one had some concerns, suggesting that most of the studies had reported pre-planned outcome measures and the selectivity of reporting was less (Figure 11). For domain 6 (overall bias), most of the studies had a high RoB (n=21), three had some concerns [23,25,28], and two studies [41,24] had a low RoB (Figure 10 and Table 3). A major chunk of bias occurred in measuring the outcome domain (Figure 11).
